# Single-cell analysis of skin and blood reveals systemic immune responses to ultraviolet B and their impairment in MS

**DOI:** 10.1186/s12974-026-03887-z

**Published:** 2026-06-03

**Authors:** Simon Falk, Julia Krämer, Sonja Leson, Davide Pilo, Marie Deffner, Patrick Ostkamp, I-Na Lu, Tilman Schneider-Hohendorf, Andreas Schulte-Mecklenbeck, Christian Wünsch, Catharina C. Gross, Kerstin Steinbrink, Antje Bischof, Catharina Korsukewitz, Gerd Meyer zu Hörste, Luisa Klotz, Carsten Weishaupt, Heinz Wiendl, Nicholas Schwab

**Affiliations:** 1 Department of Neurology, University of Münster, Albert-Schweitzer-Campus 01, Münster, 48149 Germany; 2 Department of Dermatology, University of Münster, Münster, Germany; 3 Department of Dermatology, Venereology, and Allergology, Charité - Universitätsmedizin Berlin, Berlin, Germany; 4 Department of Neurology and Neurophysiology, University of Freiburg, Freiburg, Germany

## Abstract

Sunlight is a fundamental immune-modulating factor with implications for the pathogenesis of autoimmune diseases such as multiple sclerosis (MS). MS incidence and severity increase with latitude, historically attributed to diminished ultraviolet (UV) exposure and lower serum vitamin D (VitD) levels. While high-level VitD supplementation yields moderate clinical benefit in early MS, the broader immunomodulatory potential of UV radiation remains poorly understood. To further investigate this effect on the immune transcriptome, we conducted a clinical trial (NCT05627609) in which healthy donors (HD) and MS patients were irradiated with narrowband UVB, followed by single-cell transcriptomic analysis of immune cells from the skin, blood, and cerebrospinal fluid (CSF). We revealed profound UV effects not only in the skin but also in peripheral blood and, preliminarily, in CSF. These included immune-modulating and migratory signatures across multiple cell populations, focusing on UVB triggered re-circulating myeloid- and CD8⁺ T cells, revealing a blunted translation from skin to blood in MS. Patients showed an exaggerated cutaneous immune activation with elevated stress-responsive genes (*NR4A1*,* JUN*,* HSPA1B*), while HD showed stronger induction of homeostatic and regulatory genes (*EEF1A1*,* TPT1*,* TMSB4X*) in blood. This uncovers a dysfunctional axis of immunoregulation in MS and reveals the immuno-landscape underlying the latitude-associated MS risk.

**Supplementary Information:**

The online version contains supplementary material available at 10.1186/s12974-026-03887-z.

## Introduction

Multiple sclerosis (MS) is a chronic inflammatory and neurodegenerative autoimmune central nervous system (CNS) disease that affects approximately 2.8 million people globally [1]. Characterized by both focal inflammatory lesions and diffuse, low-grade inflammation throughout the brain and spinal cord, MS leads to progressive axonal and neuronal loss, resulting in a wide spectrum of clinical symptoms, cognitive impairment, and long-term disability [2, 3].

While the precise etiology of MS remains unclear between genetic and environmental factors, the latter are thought to contribute up to 80% of the risk for the onset of MS [4, 5]. The Epstein-Barr virus has convincingly been shown to be a necessary, but not sufficient prerequisite to develop MS [6, 7]. The latitude effect of one prominent environmental factor, sunlight, is visible on a worldwide scale [1, 8]. Moreover, it has been known since the 1960s that the risk of developing MS increases with latitude, presumably due to less ultraviolet (UV) radiation reaching the skin [1, 8–10]. Furthermore, living at lower latitudes was shown to be associated with reduced disease severity as well as higher Vitamin D (VitD) serum levels [9, 11]. UV radiation (UVR) has been shown to exert direct immunological effects that may be relevant to MS pathogenesis [12, 13]. While UVC(< 280 nm) is absorbed and scattered in the atmosphere, UVA (320–400 nm) and UVB (290–320 nm) reach the earth’s surface [14]. Between UVA and UVB, the former penetrates the dermis while the latter is absorbed in the epidermis [15]. UVB absorption can modulate the innate and adaptive immune system by influencing antigen-presenting cells, regulatory T cells (Tregs), and inflammatory cytokine profiles [16–18]. VitD is considered to be one of the most important contributors to these changes [19]. In the skin, provitamin 7-dehydrocholesterol (7-DHC) absorbs UVB and is metabolized further by hydroxylation in the liver and in the kidneys. It then exerts its function in the active form 1-α, 25-dihydroxy vitamin D3 [1α,25(OH)2D3] by binding the Vitamin D Receptor (VDR) [20]. The VDR is expressed by various immune cells and modulates downstream signaling upon binding [21]. As such, VitD was in focus of recent research regarding the question of a causative effect of high latitude to trigger MS and whether supplementation might be beneficial to counteract or prevent the development of MS symptoms [21, 22]. Although favorable changes in oxidative-stress pathways were proven, data from clinical studies are still inconclusive [23–25]. Prior literature is, therefore, consistent with the hypothesis that there are VitD-dependent and –independent beneficial effects of UVB influencing MS development and disease course [25–31]. Studies in animal models of MS, such as experimental autoimmune encephalomyelitis (EAE), suggest that UVB exposure reduces neuroinflammation and alters immune cell trafficking independent of VitD [32, 33]. In humans, it was shown that among other lineages macrophages increased in proportion, and CD8 and CD4 T cells were reduced after a single UVB irradiation [34]. Tissue-resident memory (TRM) T cells are of special interest in cutaneous immunity as they take up a sizable portion of the adaptive immune surveillance and continuously circulate between skin and blood [35–38]. These cells help maintain barrier homeostasis and regulate systemic tolerance. Importantly, CD8 T cells, both regulatory and effector, are critical to this equilibrium and also implicated in CNS autoimmunity; they densely populate MS lesions, exhibit clonal expansion, and contribute to disease progression [39–44].

However, the mechanisms by which UVB irradiation alters the circulation, tissue homing, and function of CD8⁺ T cells across skin, blood, and CSF, particularly in individuals with MS, remain poorly understood.

In the current study (ClinicalTrials.gov NCT05627609), we investigated the immunological effects of controlled clinical UVB irradiation in both relapsing-remitting MS (RRMS) patients and healthy donors (HD) by analyzing immune cell populations in the skin, peripheral blood, and cerebrospinal fluid (CSF). By examining these compartments on single-cell resolution, we aimed to determine if and in what manner UVB induces immune modulation in the skin and whether this effect is expanded to systemic and CNS-associated immune cells. Specifically, we assessed changes in immune cell phenotypes, cytokine expression, and migratory patterns following UVB exposure. Given that MS is characterized by dysregulated immune responses and altered CNS immune surveillance, understanding the impact of UVB irradiation on these compartments may provide new insights into the potential therapeutic role of UVB-mediated immunomodulation in MS.

This is the first study to directly examine skin and systemic immune responses to controlled, medical-grade UVB exposure on a single-cell resolution in patients, as well as healthy individuals. The data from this study will facilitate novel insights into how local UVB-induced changes in the skin influence peripheral- and central immunity.

## Results

### UVB-induced changes of immune cell proportions in skin and peripheral blood differ between HD and MS

**Fig. 1:**
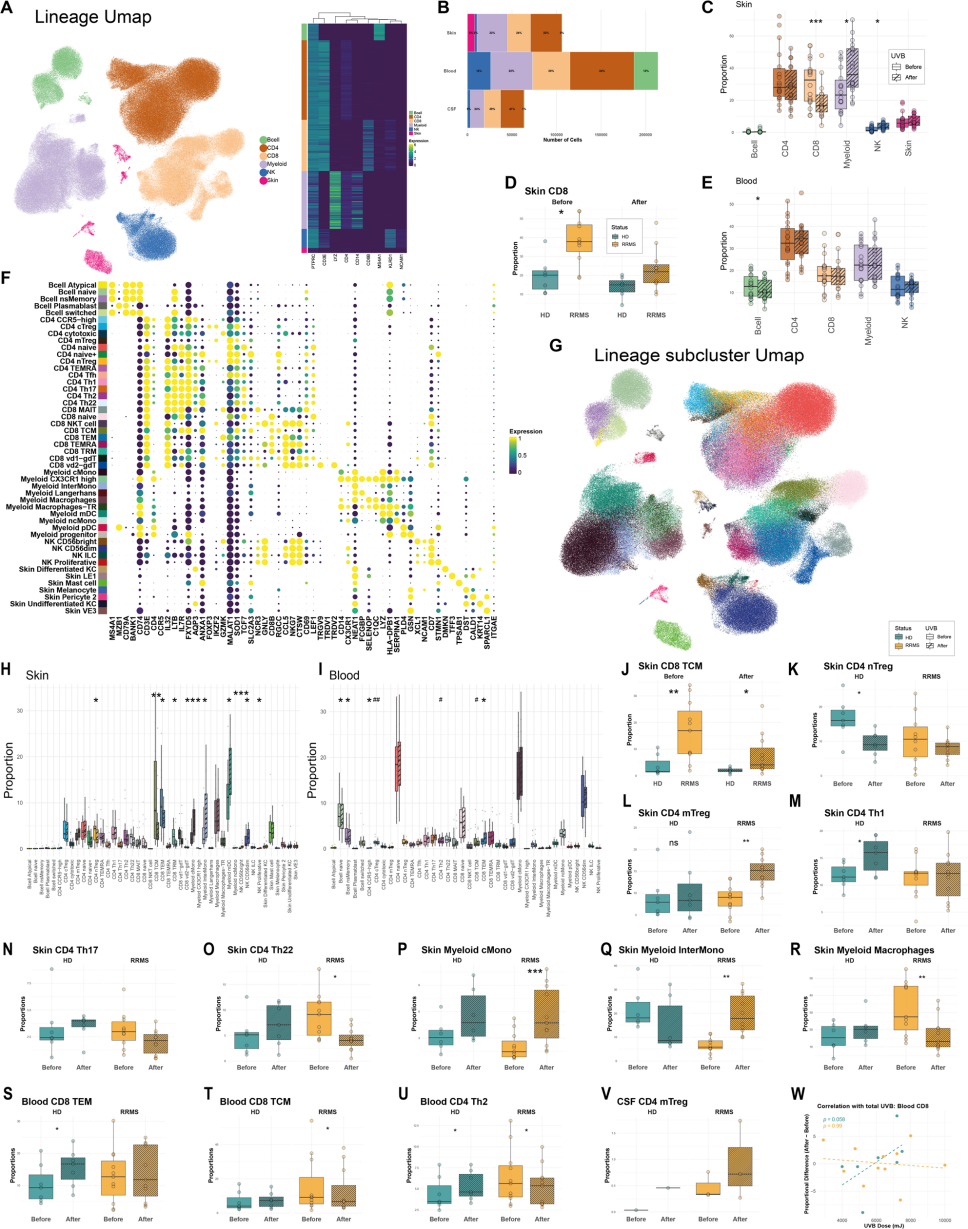
UVB-induced changes of immune cell proportions in skin and peripheral blood differ between HD and MS. **A** Lineage clusters of immune cells from skin, blood, and CSF of HD (*n*=31) and RRMS (*n*=51) visualized with UMAP and heatmap of lineage cells (*n*=384399) and marker. Skin lineage consists of only in skin found celltypes, annotated by CellTypist (v1.6.2). **B** Absolute cell counts per lineage across tissues. Stacked bar plot depicting the number of cells belonging to each lineage in CSF (*n*=10), blood (*n*=36), and skin (*n*=36). Percentages indicate relative proportions within each tissue. **C** Box plots depicting proportional changes in lineages before and after UVB in skin (*n*=36). Boxplots show the median (center line), interquartile range (box), and whiskers extending to 1.5× IQR; points beyond are outliers. **D** Box plot depicting proportional change of CD8 lineage in HD (*n*=7) and RRMS (*n*=11) skin before and after UVB irradiation. Boxplots show the median (center line), interquartile range (box), and whiskers extending to 1.5× IQR; points beyond are outliers. **E** Box plots depicting proportional changes in lineages before and after UVB in blood (*n*=36). Boxplots show the median (center line), interquartile range (box), and whiskers extending to 1.5× IQR; points beyond are outliers. **F** Dot heatmap of gene expression across cell groups. Rows represent cell groups ("fin"), and columns represent selected genes. Dot color intensity indicates the scaled average gene expression (z-score), while dot size represents the percentage of cells expressing the respective gene within each group. G, Lineage subclusters of immune cells from skin, blood, and CSF visualized with UMAP. **H**, **I** Box plots depicting proportional changes in lineage subcluster before and after UVB in skin (*n*=36) and blood (*n*=36). Boxplots show the median (center line), interquartile range (box), and whiskers extending to 1.5× IQR; points beyond are outliers. **J**-**U** Box plots depicting proportional changes of lineage and lineage subcluster in HD (*n*=7) and RRMS (*n*=11) skin and blood. Boxplots show the median (center line), interquartile range (box), and whiskers extending to 1.5× IQR; points beyond are outliers. **V** Box plot depicting proportional change of CD4 memory regulatory T cell in HD (*n*=1) and RRMS (*n*=3) CSF before and after UVB irradiation. Paired Wilcoxon signed-rank tests were used to assess within-group differences in proportions between UVB conditions and resulting p-values are displayed as significance annotations (* =*p* < 0.05, **=*p* < 0.01, ***=*p* < 0.001), significant interactions between status (HD&RRMS) are marked by # (# = *p* < 0.05, ## =*p*<0.001). Boxplots show the median (center line), interquartile range (box), and whiskers extending to 1.5× IQR; points beyond are outliers. **W** Scatter plot depicting the differences in CD8 proportions between before and after UVB irradiation in HD (*n*=7) and RRMS (*n*=11) blood against the total UVB irradiation received in mJ, Spearman correlation coefficients were calculated separately for each status group; dashed lines represent linear regressions fitted per group

Single-cell RNA sequencing was performed on isolated immune cells of skin, blood, and CSF from 11 RRMS and 7 healthy participants, before and after four weeks of UVB irradiation (Table 1) (Supplementary Table 1). After quality control, 384,399 cells were integrated and clustered based on their gene expression (Fig. 1A-B). At baseline, CD4 T cells were the largest proportion of immune cells in each of the three compartments while B cells only had a sizable population in the blood (Fig. 1B) (Supplementary Fig. 1). We combined cells from both HD and MS participants for an analysis of UVB influence in the different compartments for B cells, CD4 and CD8 T cells, myeloid cells, natural killer (NK) cells and skin cells (Fig. 1C-E), their subclustered cell types (Fig. 1F-I), and additionally investigated cellular subtypes separately by compartments (Supplementary Fig. 2–4). In skin, CD8 T-cell proportions were reduced by UVB irradiation (*p* < 0.001) (Fig. 1C), while myeloid- and NK-cell proportions increased (*p* < 0.05). Before UVB irradiation, the CD8 T-cell proportion was higher in RRMS compared to HD (Fig. 1D). In detail (Fig. 1J-V), skin CD8 T cells contained more central-memory T cells (TCM) of total cells in RRMS patients (CD8 HD TCM: HD 2% versus RRMS 17%, *p* = 0.0041) before UVB irradiation (Fig. 1J). Both percentages of CD8 TCM and effector-memory T cells (TEM) were reduced after UVB irradiation (CD8 TCM: before UVB 8% versus after UVB 3%, *p* = 0.0019; CD8 TEM: before UVB 9% versus after UVB 6%, *p* = 0.034) (Fig. 1H). Even with the decrease of CD8 TCM, the proportion remained higher in RRMS after intervention (CD8 HD TCM: HD 2% versus RRMS 4%, *p* = 0.015) (Fig. 1J). For CD4 T cells, the proportion of naïve Tregs of total cells in skin was reduced after UVB irradiation (4% vs. 2%, *p* = 0.03) (Fig. 1H), but only in RRMS patients’ memory Treg proportions increased among CD4 T cells (HD: 8% vs. 8%; *p* = 0.9; MS: 9% vs. 15%; *p* = 0.007) (Fig. 1K). Concerning other CD4 T-cell subpopulations, Th1, Th17 and Th22 proportions increased in HD (Th1: 11% vs. 16%, *p* = 0.047; Th17: 2% vs. 4%, *p* = 0.6; Th22: 5% vs. 7%, *p* = 0.5), but were nominally or significantly reduced in RRMS patients after UVB irradiation (Th17: 3% vs. 2%, *p* = 0.06; Th22: 9% vs. 4%, *p* = 0.02) (Fig. 1M-O). Myeloid cell percentages were strongly increased in the skin after UVB irradiation (before UVB 23% vs. after UVB 36% *p* = 0.018) (Fig. 1H), mainly driven by the increase of classical monocytes in RRMS (HD: 3% vs. 5%, *p* = 0.16; RRMS: 1% vs. 5%, *p* = 0.00098) and of intermediate monocytes in RRMS (before UVB 1% vs. after UVB 9%, *p* = 0.002), which was accompanied by a decrease of macrophages of myeloid cells in this group (before UVB 27% vs. after UVB 13%, *p* = 0.002) (Fig. 1P-R). The increase of NK cells (*p* < 0.05) was mainly driven by CD56 bright cells of total cells (before UVB 0.09% vs. after UVB 0.39%, *p* = 0.0002) (Fig. 1H).

Changes in immune cell composition were not only detectable in skin but also in peripheral blood with a significant decrease of B-cell proportions (before UVB 13% vs. after UVB 10%, *p* = 0.02) (Fig. 1E), mainly driven by a reduction of naïve B cells (before UVB 7% vs. after UVB 6%, *p* = 0.002) (Fig. 1I & Supplementary Fig. 5). Additionally, CD8 TEM percentages of total cells increased after UVB irradiation (before UVB 1.7% vs. after UVB 2.3%, *p* = 0.048). In detail, this shift is most present in HD (Fig. 1S & Supplementary Fig. 5), accompanied by an increase in CD4 Th2 of CD4 T cells in HD (Fig. 1U ), while there is a decrease in RRMS of CD8 TCM and CD4 Th2, as was validated with flow cytometry (HD: 3.5% vs. 4.6%, *p* = 0.016; RRMS: 5.6% vs. 5.4%, *p* = 0.032) (Fig. 1T-U & Supplementary Fig. 5). In the CSF, no significant changes were observed, but memory Tregs nominally increased (before UVB 0.08% vs. after UVB 0.22%, *p* = 0.6) in RRMS patients with a similar trend in HD (Fig. 1V). Interestingly, the changes in proportions of CD8 T cells positively trend with the total amount of UVB irradiation, specifically in HD (*p* = 0.058 for change in HD) (Fig. 1W& Supplementary Fig. 5).

**Table 1:** Overview of collected data

													Vitamin D Serumlevels (ng/mL)					
	Sex		Age		Skin		Blood		CSF		UVB		Baseline		After UVB		Delta	
Status	Male	Female	Median	Mean	Baseline	After UVB	Baseline	After UVB	Baseline	After UVB	Median	Mean	Median	Mean	Median	Mean	Mean	Mean
HD	3	4	29	39.1428571428571	16883(n=7)	35306(n=7)	38335(n=7)	40116(n=7)	14818(n=2)	875(n=1)	6250	6014.28571428572	23.4	21.0857142857143	39.4	36.6428571428571	15.1	15.5571428571429
RRMS	6	5	42	43.9090909090909	25656(n=11)	28562(n=11)	75745(n=11)	59751(n=11)	27484(n=5)	20868(n=3)	6400.00	6237.27272727273	25.8	23.9454545454545	34.5	32.7909090909091	7.8	8.84545454545454

Metadata of the two disease status groups HD and RRMS, including the number of cells and Vitamin D 25(OH) serum levels.

### UVB-induced gene expression changes in peripheral blood are more pronounced in healthy donors

**Fig. 2:**
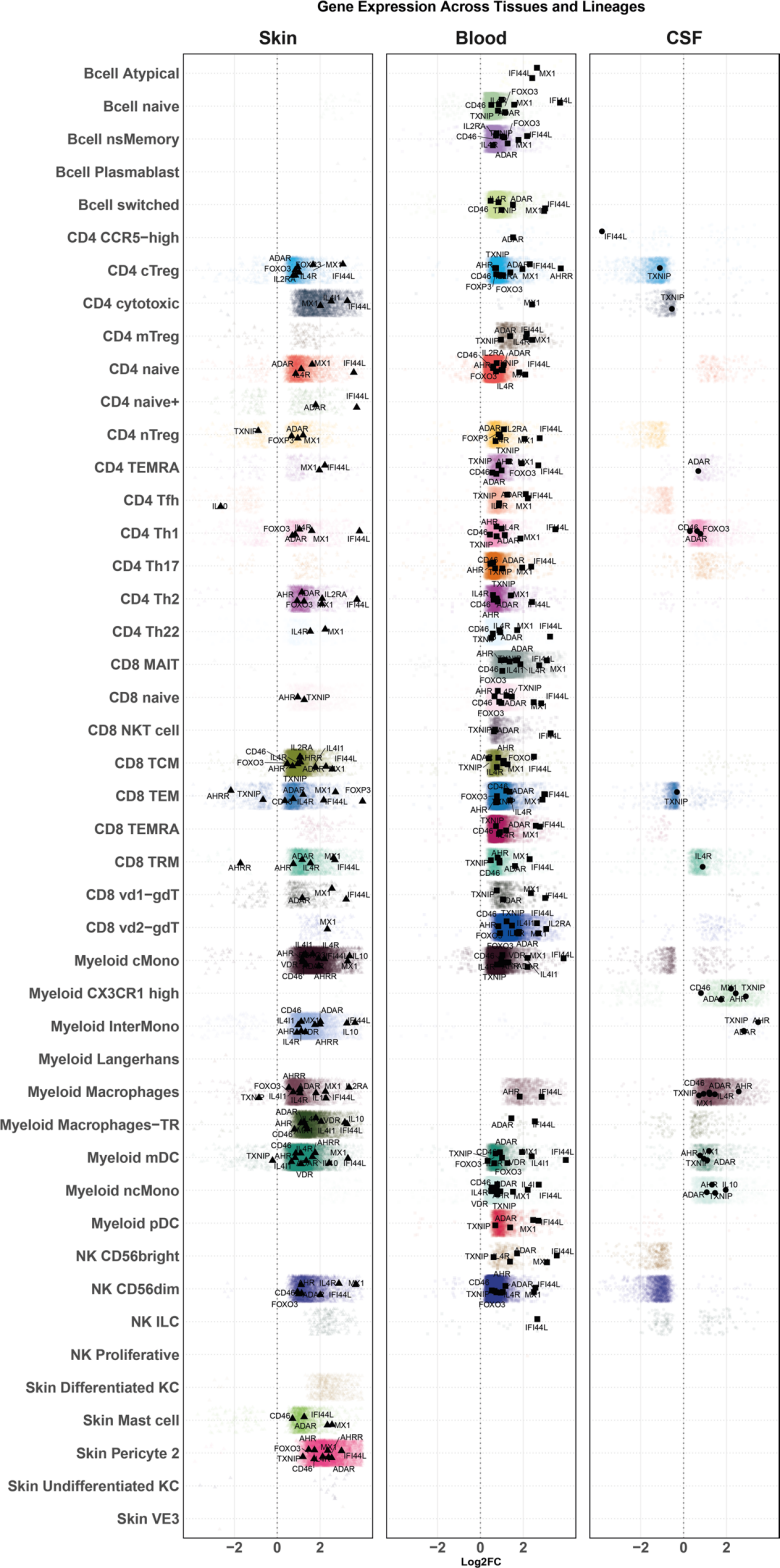
Differentially expressed genes overview. **A** Log₂FC in gene expression after UVB irradiation for all genes significantly differentially expressed (adjusted *p*-value < 0.05) as determined by the NBMLM tool nebula. Data are shown for skin (*n* = 36), blood (*n* = 36), and cerebrospinal fluid (CSF; *n* = 10). Y-axis was “center-squished”: magnitudes ≤ 2 were linearly stretched two-fold, while larger magnitudes were sign-preserving and logarithmically compressed

**Fig. 3:**
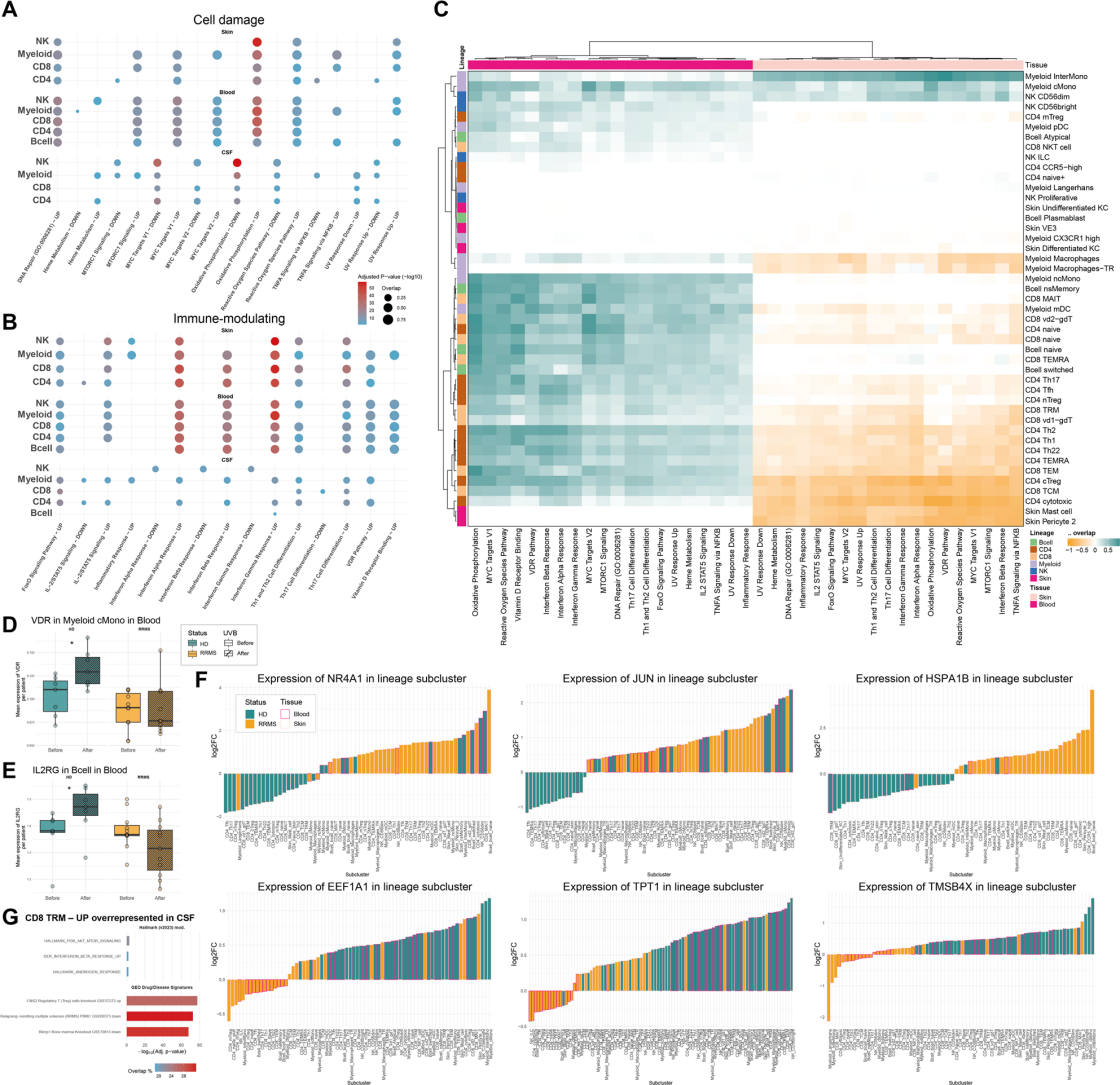
After UVB irradiation changes in gene expression in peripheral blood are more pronounced in healthy donors. **A**, **B** Dot plot of overrepresented pathways among significantly changed gene expressions (adjusted *p*-value < 0.05) after UVB irradiation in lineages across skin, blood, and CSF. **C** Heatmap of significant (*p* <0.05) differences, as determined by Fisher’s exact test, in overlap of only upregulated overrepresented pathways between HD and RRMS (HD-RRMS) in all lineage subclusters from skin and blood clustered with ward.D2. **D** Box plot depicting mean expression of VDR in classical monocytes in HD (*n*=7) and RRMS (*n*=11) blood before and after UVB irradiation. Boxplots show the median (center line), interquartile range (box), and whiskers extending to 1.5× IQR; points beyond are outliers. **E** Box plot depicting mean expression of IL2RG in B cells in HD (*n*=7) and RRMS (*n*=11) blood before and after UVB irradiation. Boxplots show the median (center line), interquartile range (box), and whiskers extending to 1.5× IQR; points beyond are outliers. **F** Gene expression log2 Fold-Changes as determined by nebula analysis for HD and RRMS separately across different lineage subclusters for different genes, ranked among the top 10 based on a composite delta metric reflecting opposite-directional shifts between groups. **G** Overrepresentation analysis among significantly upregulated genes in RRMS CSF-derived CD8 TRM cells. The bar plots display the top 3 significances. Bar length corresponds to overrepresented significance (−log₁₀ adjusted p-value), while bar color intensity represents the overlap percentage of genes within each pathway

Differential gene expression analysis was performed with a negative-binomial mixed model to explicitly account for subject-level random effects while maintaining cell-level information. Thus, over 200,000 instances of significant changes (*p* < 0.05) in gene expression levels through 48 lineage subclusters across three compartments were revealed (Fig. 2). Consistent expression changes were identified across cell populations, such as for the two genes *TXNIP* and *FOXO3*. *TXNIP*, thought to be negatively correlated with MS, was increased in 29 different lineage subclusters in blood. Elevated expression levels of *FOXO3* were shown to be associated with the pathogenesis of autoimmune diseases and were upregulated (*p* < 0.05) in 23 different instances across compartments and lineage subclusters after UVB irradiation. Also, *FOXP3* was upregulated (*p* < 0.05) after UVB irradiation in naïve and cytotoxic Tregs in the skin, and naïve Tregs in blood (Fig. 2) (Supplementary Fig. 6). Interferon-associated genes such as *IFI44L*, *MX1*, or *ADAR* were upregulated in at least 50 different instances. Those changes were apparent in an analysis of overrepresented genes (Fig. 3A-B). As expected, genes associated with heme metabolism, oxidative phosphorylation, and DNA repair were upregulated (*p* < 0.05) among all lineages, except B cells, most probably due to their low prevalence in the skin (Fig. 3A). Importantly, those changes were not only detectable in the skin but were also upregulated (*p* < 0.05) in the blood and even showed activity among the genes of Oxidative Phosphorylation and Reactive Oxygen Species pathway in CSF (*p* < 0.05). Inflammation-associated pathways like TNF-alpha signaling via NF-kappaB and mTORC1 were also enhanced (*p* < 0.05) after UVB irradiation among all lineages in skin and blood, except in B cells. Furthermore, exploratory analysis showed a decrease in CSF on some lineages, most notably in NK cells concerning the mTORC1 pathway and in myeloid and CD4 T cells concerning TNF-alpha signaling via NFK-kappaB, for which we observed no upregulation only in CD4 T cells in the CSF.

In addition to cell damage-associated pathways, immune-modulating genes were enriched as well (Fig. 3B). The VDR pathway was upregulated (*p* < 0.05) in CD8, CD4 T cells, and in myeloid cells in skin and in all lineages of the blood. In CSF, it was also elevated in myeloid cells and CD4 T cells. Interferon pathways were universally upregulated among all lineages in skin, blood, and, albeit less pronounced, in myeloid cells and CD4 T cells in CSF. Lastly, pathways promoting Th1 and Th2, as well as Th17 cell differentiation, were overrepresented (*p* < 0.05) in all three compartments.

Although the modulated pathways due to UVB irradiation were similar in HD and RRMS participants, the intensity of these changes strongly differed between the two groups. To compare UVB-responsive pathways between groups, we performed over-representation analysis separately in RRMS and HD for each tissue and cell type. For every gene set, we calculated the fraction of set members that were differentially expressed after UVB in that group (FDR < 0.05), then computed the between-group difference (HD-RRMS). Positive values indicate a larger share of genes changing in RRMS, and negative values indicate the reverse. Percentages of cell damage-associated gene sets were higher in HD blood, predominantly in CD4 and CD8 T cells, but also in myeloid cells such as classical and non-classical monocytes and dendritic cells, and most B-cell types such as switched and non-switched, as well as the major NK cell populations CD56-dim and CD56-bright (Fig. 3C). Oxidative Phosphorylation showed the biggest differences in blood between HD and RRMS and clustered most outward, while TNFA signaling via NFKB, UV Response (Down) and Inflammatory Response presented with smaller differences between HD and RRMS, and clustered closest to all pathways in the skin. Unsupervised clustering of the values separated skin and blood into distinct clusters, consistent with compartment-specific responses. Across cell types in the skin, Δ-fractions were generally negative, highlighting larger UVB-induced transcriptional modulation in RRMS skin compared with HD, whereas blood showed opposite shifts. In cytotoxic CD4, cytotoxic Tregs CD4, and CD8 TCM genes of all pathways were most strongly overrepresented in RRMS participants. CD8 TEMRA showed a slightly higher representation of Interferon pathways in HD and in CD56-dim NK cells, whereas for classical and intermediate monocytes, all pathways were more pronouncedly represented in HD participants. Even though RRMS participants showed a much stronger gene pathway modulation by UVB irradiation in the skin, those changes did not translate to immune cells in peripheral blood, where the changes were much more pronounced in HD. As examples of this common pattern, the VDR gene in classical monocytes and IL2RG in B cells increased in HD blood but remained unchanged in RRMS patients (Fig. 3D–E). Supporting this pattern, stress-responsive genes such as *NR4A1*, *JUN*, and *HSPA1B* were upregulated predominantly in the skin of RRMS patients, while homeostatic and translational genes, including *EEF1A1*, *TPT1*, and *TMSB4X*, showed stronger induction in blood-derived lineage subsets from HD but not RRMS (Fig. 3F). Notably, these genes ranked among the top differentially modulated targets based on combined directional shifts across groups, highlighting their prominence in UVB-driven immune divergence (Supplementary Table 2). These findings emphasize a compartmentalized and disease-modified UVB response, with HD favoring systemic maintenance and RRMS favoring local stress adaptation (Fig. 3F; Supplementary Table 2). Despite the blunted systemic response in MS, UVB-induced expression changes reached the CSF (Fig. 3A-B). Among significantly upregulated genes in CD8 TRM, Interferon-β response and regulatory T-cell expression patterns were overrepresented (Fig. 3G). Additionally, genes previously reported as downregulated in RRMS patient PBMCs were notably upregulated, suggesting adjustments in immune regulation. Furthermore, when analyzing vitamin D-isolating transcriptional changes, gene overrepresentation was predominantly observed among downregulated pathways (Supplementary Fig. 7A&B). This downregulation was most pronounced in CD4⁺ T cells from HD blood, while in RRMS it was skewed toward CD8⁺ T cells, indicating a shift in the compartment and cellular context of vitamin D-responsive immune modulation (Supplementary Fig. 7C).

### Translation of skin-induced changes in cell-cell communication after UVB into peripheral blood is dysfunctional in MS

**Fig. 4:**
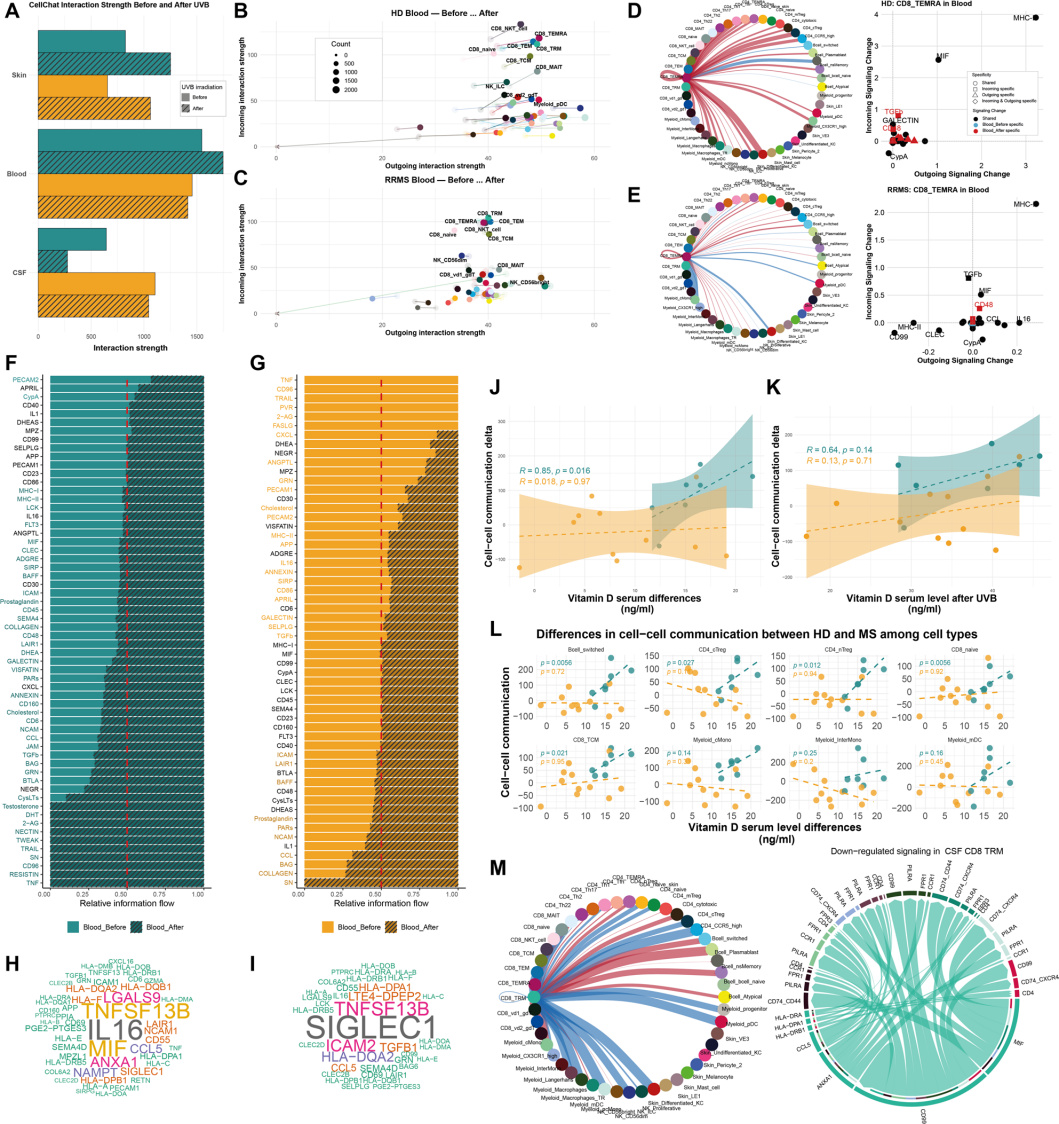
Translation of skin-induced changes in cell-cell communication after UVB into peripheral blood is dysfunctional in MS. **A** The strength of inferred interactions as calculated by CellChat from before and after UVB irradiation in skin, blood, and CSF of HD and RRMS. **B**, **C** Scatter plots illustrating the signaling roles (incoming vs. outgoing interactions) of blood-derived cells before and after treatment. Data are shown separately for healthy donors (HD, **B**) and relapsing-remitting multiple sclerosis (RRMS, **C**) patients (bottom row), the ten cell types with highest combined signaling strength in the “After” state are labeled. **D**, **E** Comparative signaling analysis of CD8 TEMRA cells in blood samples. Left panel showing chord diagram with differential cell-cell interactions involving CD8 TEMRA cells in HD (**D**) and RRMS (**E**). Right panel depicts scatter plot illustrating changes in outgoing and incoming signaling between before and after UVB. **F**, **G** Stacked bar plot showing the weight in the relative information flow of signaling pathways before and after UVB irradiation in HD (**F**) and RRMS (**G**), processed with CellChat and significant differences highlighted in colored text as calculated by a paired Wilcoxon test. H, **I** Wordcloud showing most enriched receptor and ligand genes after UVB irradiation as calculated by CellChat in HD (**H**) and RRMS (**I**). **J** Scatter plot depicting the differences in cell-cell interaction per patient between before and after UVB irradiation in HD (*n*=7) and RRMS (*n*=11) blood against differences in VitD serum levels before and after UVB,  lines represent linear regressions fitted per group. **K** Scatter plot depicting the differences in cell-cell interaction per patient between before and after UVB irradiation in HD (*n*=7) and RRMS (*n*=11) blood against the VitD serum level after UVB in ng/ml, lines represent linear regressions fitted per group. **L** Scatter plots depicting the differences in cell-cell interaction per patient in lineage subclusters between before and after UVB irradiation in HD (*n*=7) and RRMS (*n*=11) blood against the VitD serum level after UVB in ng/ml ,  Spearman correlation coefficients were calculated separately for each status group, dashed lines represent linear regressions fitted per group. **M** Comparative signaling analysis of CD8 TRM cells in CSF samples. Left panel showing chord diagram with differential cell-cell interactions involving CD8 TRM cells in RRMS CSF before and after UVB (*n*=3). Right panel shows a chord diagram illustrating downregulated signaling interactions from CD8 TRM cells to myeloid cell populations. Connections represent signaling pathways significantly reduced in CSF CD8 TRM after UVB. Interaction strength is indicated by chord thickness

To set the observed changes in gene expression into a cellular context, we analyzed cell-cell communication among the 48 cell types regarding UVB irradiation with CellChat (Fig. 4A-M). CellChat infers cell–cell communication by using single-cell RNA-seq ligand–receptor expression to estimate signaling probabilities between cell types, grouping interactions into pathways and assessing significance via permutation. Expectedly, the total number of interactions between lineage subclusters as well as their strength increased in both HD and RRMS in the skin by UVB irradiation (Interactions: HD 58% vs. RRMS: 49%) (Fig. 4A) (Supplementary Fig. 8). However, this increase only translated to an increase in HD blood with an unexpected decrease in RRMS blood (HD: 11%; RRMS: -6%). Among all lineage subclusters in blood, CD8 T cells were strong communicators not only in outgoing interaction strength but especially in incoming interaction strength (Fig. 4B-C). While we saw an increase of most lineage subclusters in HD blood, shifts were rare in RRMS blood and partly decreasing like NK CD56 - dim and bright. One of the strongest communicators in HD blood were CD8 TEMRA, increasing *MIF*, *MHC-I*, and *TGFb* signaling strength among almost all lineage subclusters, except strongly decreasing to plasmablasts (Fig. 4D). In contrast, in RRMS CD8 TEMRA, increases in signaling strength were less pronounced and even decreased in *CD99*, *MHC-II* and *CLEC* signaling (Fig. 4E). This was further substantiated when comparing the relative information flow of signaling pathways before and after UVB intervention (Fig. 4F-G). Pathways that were weighted higher (*p* < 0.05) after UVB dominated for HD in blood, whereas in RRMS blood, they were weighted higher before UVB irradiation. *CCL* and *NCAM* signaling were weighted higher after UVB irradiation among HD and RRMS. On the contrary, the *CD96* pathway, important for NK- and T-cell communication, and the *TNF* pathway were weighted higher after UVB in HD and before UVB in RRMS. Ligands that were enriched most, e.g. *SIGLEC1* in RRMS, were strongly modulated in myeloid cell types after UVB, in which a lack of response in UV or *VDR*-associated genes was found for RRMS (Figs. 3 and 4H-I). Ligand and receptor genes that affected this the most in HD were, for example, migration-associated genes such as *MIF*, *ANXA1*,* and ICAM1* (Fig. 4M). When correlating the differences in interaction scores per patient with the absolute amount of serum VitD levels after UVB or the total amount of UVB irradiation, the cell-cell communication was positively trending up for both but the score levels were generally higher for HD, even though both groups received similar amounts of UVB and presented with similar absolute levels of VitD after irradiation (Fig. 4J-K) (Supplementary Fig. 7D-F). However, the correlation of interaction scores with differences in VitD serum levels after UVB irradiation could only be found in HD. In MS, the correlation, if present at all, trended only slightly (Fig. 4J). Again, when correlating with the differences in VitD serum levels (before versus after UVB), most lineage subclusters’ scores were trending positively only in HD (Fig. 4L).

Even with this impaired peripheral response, a comparable reduction in signaling strength is observed in the CSF (Fig. 4A; Supplementary Fig. 8). Again, CD8 T-cell subsets, notably CD8 TRM cells, emerge as the predominant communicators. Specifically, CD8 TRM cells markedly enhance signaling toward B-cell subsets but strongly reduce signaling toward myeloid lineages (Fig. 4M). Among these, *CD99*-mediated signaling was most prominently diminished in myeloid cells, accompanied by notable decreases in *MIF* signaling toward mDCs and reductions in *ANXA1* signaling.

### Fewer T-cell clones migrate from the skin to the peripheral blood in MS patients after UVB irradiation

**Fig. 5:**
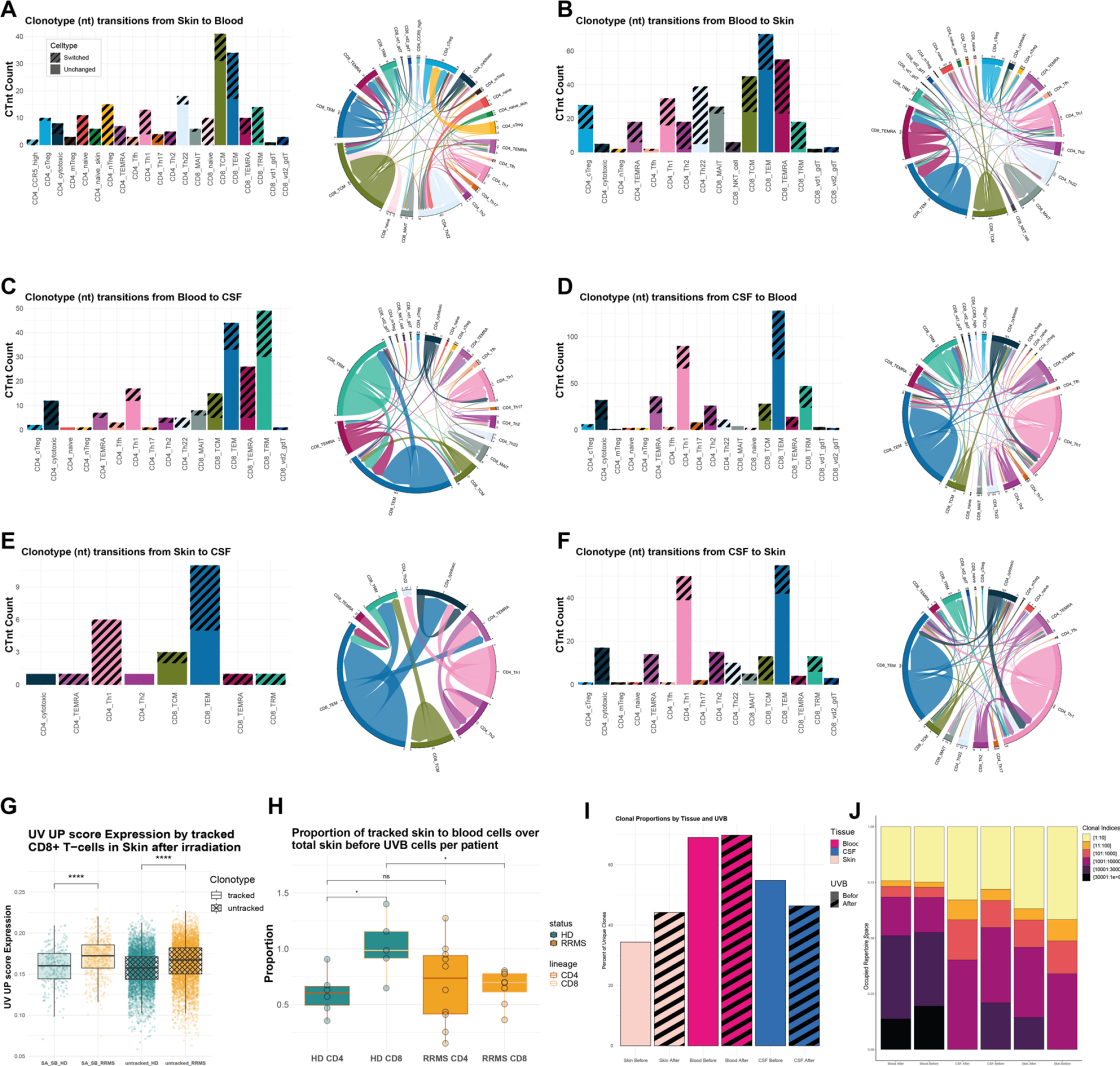
Fewer T-cell clones migrate from the skin to the peripheral blood in MS patients after UVB irradiation. **A**-**F** Stacked bar plots depicting the number of individual clonotypes defined by exact nucleotide sequence (CTnt) in T-cell lineage subclusters found expanding from one tissue before UVB irradiation to another tissue after, and whether their annotation has changed. The annotation of the found clonotypes is shown in a directional graph from before UVB irradiation to after for skin to blood (**A**), blood to skin (**B**), blood to CSF (**C**), CSF to blood (**D**), skin to CSF (**E**), CSF to skin (**F**). **G** UV Response UP, gene set of the Hallmark collection, expression scores in T cells that were tracked to stay in skin and all other after UVB irradiation in HD and RRMS compared by an unpaired Mann-Whitney U test (***=*p*-value<0.0001). Boxplots show the median (center line), interquartile range (box), and whiskers extending to 1.5× IQR; points beyond are outliers. **H** Proportions of CD4 and CD8 T cells that were found from skin to blood over total skin T cells before UVB irradiation in HD (CD4=6, CD8=5) and RRMS (CD4=10, CD8=8), per patient, compared by unpaired Mann-Whitney U test (*=*p*-value<0.05). Boxplots show the median (center line), interquartile range (box), and whiskers extending to 1.5× IQR; outliers were removed. **I** Percentages of unique clonotypes in skin, blood and CSF before and after UVB irradiation. **J** Proportions of T-cell clonotype frequencies in skin, blood, and CSF as calculated by *scRepertoire*

To build on the transcriptome analysis, T-cell receptor (TCR) sequences as well as B-cell receptor sequences (BCR) were enriched and sequenced for further investigation (Supplementary Fig. 9). Since TCR rearrangements of T-cell clonotypes are unique on a nucleotide level, clonally expanded T cells can be “tracked” between conditions and across compartments. If a clonotype was found in a tissue after UVB and was not found there before the intervention, but was found nucleotide-identically in a different tissue, it is highly likely that this clonotype infiltrated that tissue as a consequence of UVB irradiation (Fig. 5A-F). As such, we detected 119 CD8 T cell and 105 CD4 T-cell clonotypes having migrated from the skin to the blood after UVB irradiation (Fig. 5A). All naïve cell types we tracked changed their profile, such as naïve Tregs to mostly cytotoxic Tregs and naïve CD8 T cells to TCM. With 61% of all tracked clonotypes from blood to skin, again, CD8 T cells were found migrating more abundantly across compartments than CD4 T cells, which was the case for all tracings, even though we found higher CD4 T-cell percentages in each of the three tissues (Fig. 1D & Fig. 5B). CD8 TCM and TEM mostly kept their transcriptomic profile, while naïve- and some cytotoxic Tregs changed to memory-type Treg profiles. This was also observed for Tregs and CD8 memory T cells investigating tracked clonotypes from blood to CSF (Fig. 5C). Furthermore, 25 clonotypes were tracked from skin to CSF that were neither found in blood or CSF before UVB irradiation, most of which were CD4 Th1 cells or CD8 TEM (Fig. 5E).

In alignment with our observation that CD8 TEM proportions increased in the blood of HD following UVB exposure, comprising a significant fraction of tracked clonotypes, we found that HD exhibited a higher percentage of CD8 T cells tracked from skin to blood relative to total skin CD8 T cells compared to RRMS participants (HD 0.99% vs. RRMS 0.7%, *p* = 0.04) (Fig. 5G). Additionally, HD CD8 T cells residing in the skin post UVB exposure, including both tracked and non-traceable populations, displayed a higher UV-associated expression score, as defined by *A. Liberzon et al.*, than those of RRMS patients (tracked: *p* = 3.5e-11; untracked: *p* < 2.22e-16) (Fig. 5H) [45]. This discrepancy may explain the reduced amplitude of gene expression changes observed in blood post UVB in RRMS participants compared to HD.

Diversity among clonotypes also changed after UVB irradiation (Fig. 5I-J). In skin, the number of unique clonotypes increased by 29%, while in the CSF, the contrary was the case with a decrease of diversity by 14%.

### Differences in T-cell chemokine and chemokine receptor expression might contribute to reduced translation from skin to peripheral blood in MS

**Fig. 6:**
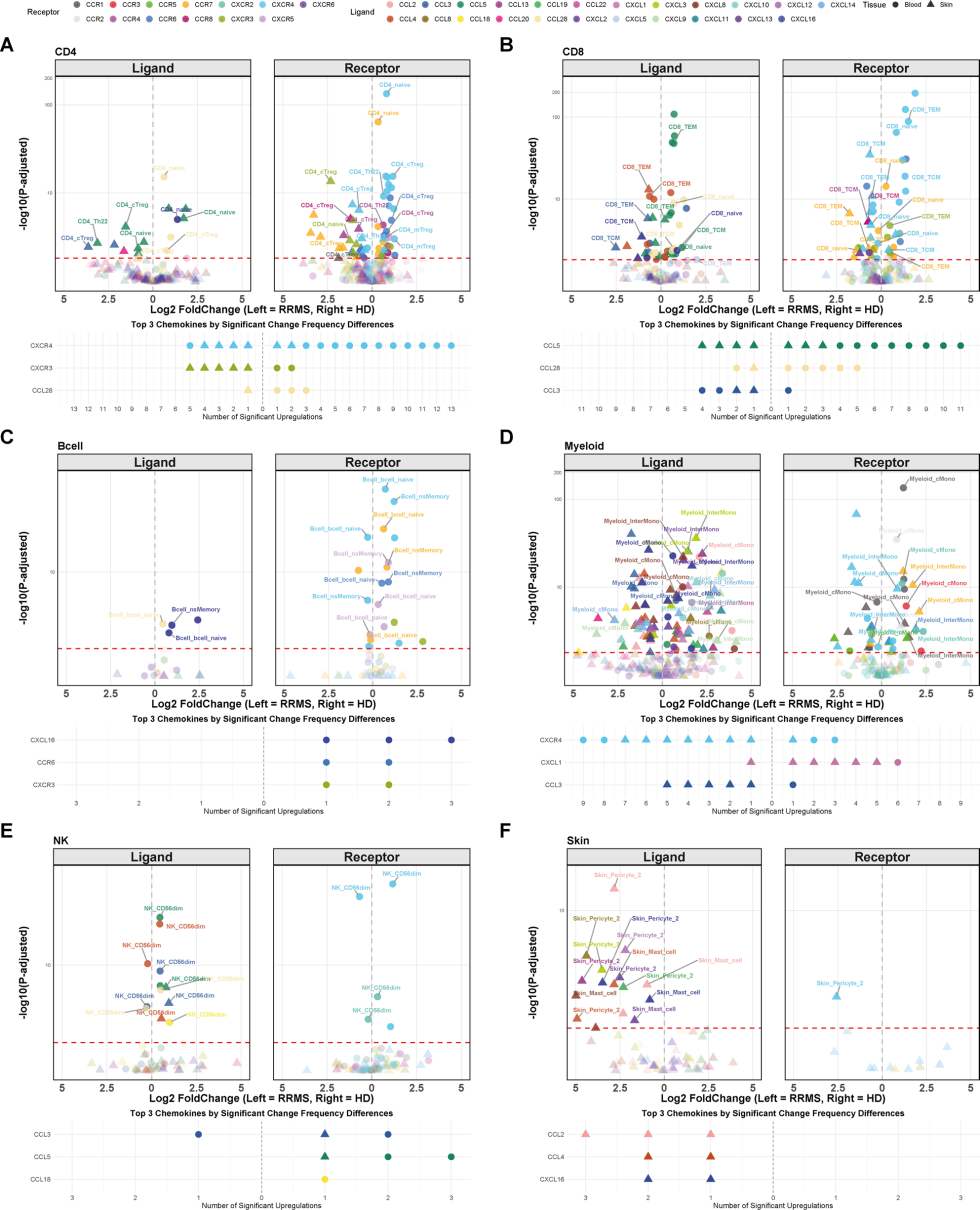
Differences in T-cell chemokine and chemokine receptor expression might contribute to reduced translation from skin to peripheral blood in MS. **A**-**F** Volcano plots displaying the Log2FC of upregulated chemokine receptors and ligands for HD and RRMS of lineage subclusters before and after UVB irradiation in CD4 T cells (**A**), CD8 T cells (**B**), myeloid (**C**), B cells (**D**), NK (**E**) and skin cells (**F**), dashed red line marks significance threshold (adjusted *p*-value < 0.05). Tissue is represented as point shape and chemokine gene as a point color, as each point depicts one instance of gene expression change. Lineage subclusters of interests were annotated

Chemokines play a vital role in cell migration and analysis of their ligand and receptor expression among leukocytes revealed differences between RRMS and HD participants. For cell types of all lineages except skin-exclusive types, we found a stronger representation of upregulated chemokines in HD blood whereas the upregulation in skin was found more often in RRMS (Fig. 6A-F) (Supplementary Fig. 10). In 11 of 12 different CD4 + T-cell types, *CXCR4* was increased (*p* < 0.01) in HD blood, but it was only increased in Th17 cells in RRMS blood, while in the skin it was upregulated in four RRMS and two HD CD4 + T-cell types (Fig. 6A). Also, *CCR7* was found in six instances of upregulation in the RRMS skin CD4 T cells but none in MS peripheral blood, where five instances were upregulated in HD. Similarly, in CD8 T cells, *CXCR4* and *CCL5* were upregulated in eight HD blood cell types, like TCM and TEM, while only in four of RRMS for *CXCR4* and none for *CCL5*, but in six of *CXCR4* in the RRMS skin vs. two in HD (Fig. 6B). In B cells, even when upregulation occurred in both groups, fold-changes were generally larger and P values smaller in HD, for example, CXCR4/CXCR5 in non-switched and naïve B cells (Fig. 6C). In myeloid cells, CXCR4 was upregulated in seven RRMS subsets versus one in HD, although chemokine induction overall was heterogeneous (Fig. 6D). Finally, in NK cells, CXCR2 showed stronger and more significant upregulation in CD56 dim NKs from HD (Fig. 6E). For skin-only cell types, we found only significant upregulations in RRMS and predominantly among ligands in pericytes (Fig. 6F). Therefore, the pattern that was already visible in cell proportions, GSEA, cell-cell communication, and T-cell migration, namely a detectable UV-induced change in immune cells in the skin of MS patients that failed to transfer to peripheral blood, was visible again in chemokine (receptor) upregulations.

## Discussion

**Fig. 7:**
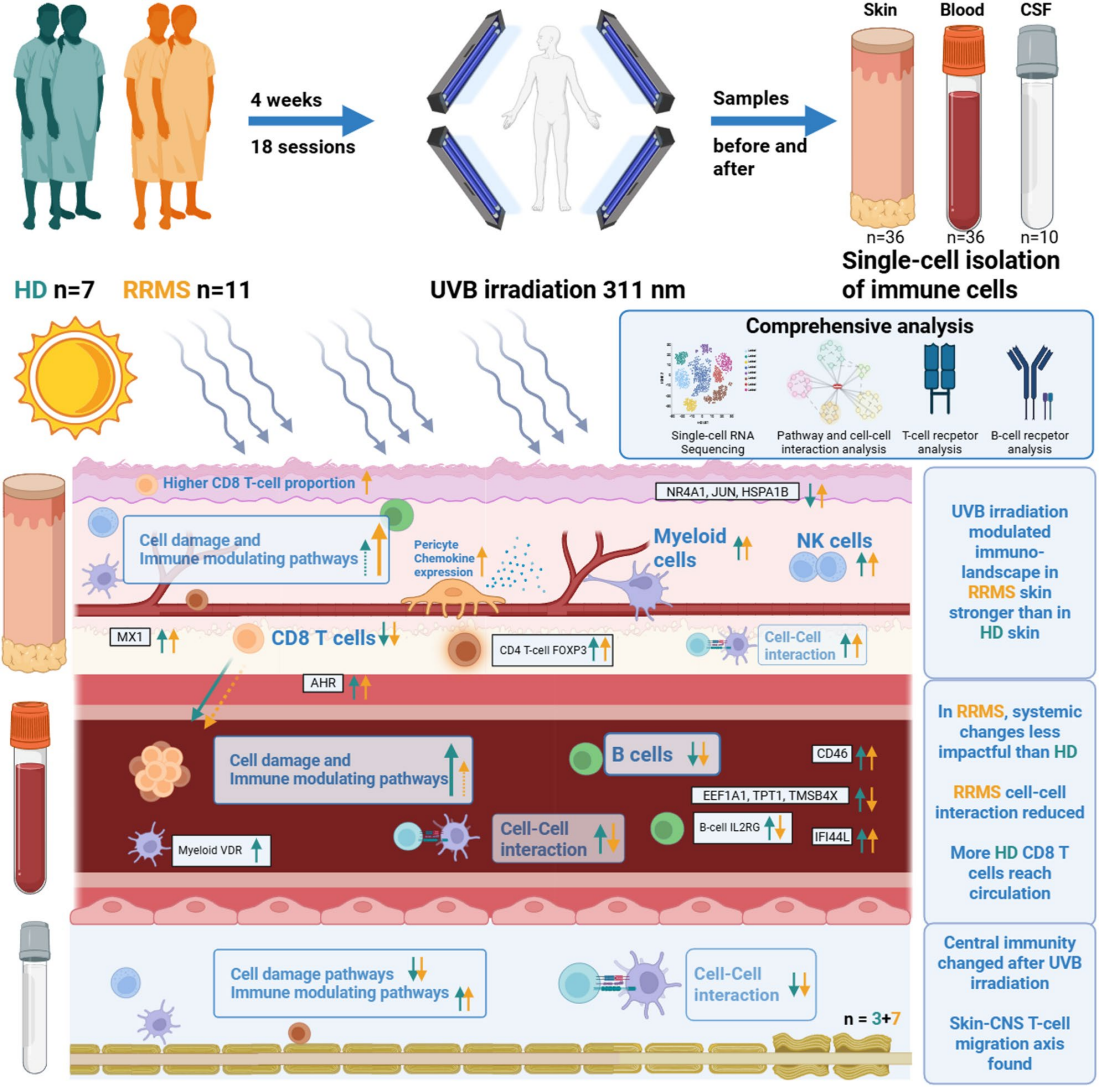
UVB-induced immune signaling propagates from skin to CSF, but is impaired in MS. Schematic of a clinical trial analyzing immune responses to narrowband UVB exposure in healthy donors and MS patients. Single-cell profiling of skin, blood, and CSF revealed compartment-spanning transcriptional changes and impaired systemic responses in MS. This figure was done with BioRender.com

In this study, we present the first investigation of the immunomodulatory effects of UVB irradiation in healthy participants and RRMS patients from the SMILE clinical trial on a single-cell resolution, specifically examining its influence on the transcriptome of immune cells in skin, blood, and CSF (Fig. 7). Through single-cell RNA sequencing, we were able to track proportional changes, migration, and transcriptional changes of immune cells across these compartments, offering a comprehensive perspective on the cellular dynamics involved in this process.

Our results demonstrate that UVB irradiation significantly reduces the proportion of CD8 T cells in the skin, a lineage that was proven to be important for cutaneous immunity [36]. Interestingly, the decline was notably more pronounced in RRMS participants. This decline predominantly involved TCMs (Fig. 1H & J), aligning with earlier findings from healthy populations indicating an acute UVB-induced response within this cell subset, which may represent a crucial regulatory mechanism to maintain skin immune homeostasis [34]. Intriguingly, at baseline, RRMS participants exhibited higher expression levels of *EIF1Y* and *USP9Y* within the overall CD8 T-cell lineage compared to HD (Supplementary Fig. 6). Previously, *EIF1Y* and *USP9Y* were characterized as Y chromosome-linked immune biomarkers expressed by CD8 T cells, generally observed at higher expression levels in the blood of healthy men rather than in male MS patients [46, 47]. Our observation thus expands on earlier studies, highlighting an unexpected enrichment of these markers within the total CD8 T-cell population in RRMS skin. However, this differential expression between RRMS and HD disappeared after UVB irradiation, suggesting that UVB exposure may selectively impact or modulate subsets within the broader CD8 lineage expressing these biomarkers.

Notably, despite larger proportional decreases of CD8 T cells in RRMS skin, fewer CD8 T cells in blood after UVB were identified as originating from the skin compartment (Fig. 5H). The recirculation of resident memory T cells is the subject of recent investigations, and this observation implies a potential defect either in the migratory capacity or survival of UVB-exposed CD8 T cells during their transition from skin into circulation in RRMS patients [48–50]. Indeed, the peripheral blood of RRMS participants displayed a diminished transcriptional activation of immunomodulatory genes, particularly among CD4 and CD8 T cells, relative to HD (Fig. 3A-C), and only HD CD8 showed a trend in increase with UVB (Fig. 1W). In contrast, skin samples from RRMS patients showed a more pronounced transcriptomic response than HD, indicating that skin-resident or infiltrating immune cells in RRMS are persistently more reactive to UVB exposure, may fail to migrate effectively into systemic circulation, or translate their transcriptional reprogramming into sustained systemic immunoregulatory effects.

Similarly, we found more increases of skin resident markers as *CD69*, *NR4A1*, and *NR4A2* in HD blood, while in RRMS, increases of such were confined to cells in the skin (Supplementary Fig. 6A&I), further supporting a decreased survival of RRMS CD8 in the skin [51]. When these UVB-affected CD8 T cells fail to re-enter circulation in RRMS, despite comparable post-UVB increases in VitD levels, RRMS CD8 T cells in blood may experience more pronounced VitD-exclusive effects, as we demonstrated (Supplementary Fig. 7C). In line with this, NR4A1, JUN, and HSPA1B, all three key stress-responsive genes, were among the most upregulated in RRMS skin, reflecting sustained local tissue activation and damage control (Fig. 3F) [52–55]. Conversely, EEF1A1, TPT1, and TMSB4X, which support translational capacity, anti-apoptotic resilience, and tissue repair, were preferentially upregulated in HD blood but remained suppressed in RRMS, highlighting the blunted systemic response to UVB [56–60]. These divergent transcriptional programs further illustrate the skewed immune plasticity in RRMS, favoring local stress adaptation over systemic homeostasis and displaying potentially interesting targets for additional drug research. Adding further complexity, we observed that pericytes, known for their pivotal role in controlling vascular permeability, leukocyte trafficking, and local immune responses, demonstrated significant transcriptional differences between RRMS and HD [61–64]. In RRMS patients, pericytes exhibited pronounced chemokine ligand expression changes and increased cellular communication networks following UVB irradiation, whereas HD pericytes either remained stable or showed reduced communication (Figs. 3C and 6F) (Supplementary Figs. 7 & 10). Notably, among the upregulated ligands in RRMS pericytes was *CXCL16*, a chemokine previously shown to orchestrate CD8 + TRM recruitment [65]. This may be one link to the impacted migration and systemic distribution of regulated immune cells post-UVB in MS. Although pericytes were not initially targeted as primary cells of interest, these results underscore their potentially important modulatory role and highlight the need for further focused analyses on their contribution to immune dynamics in RRMS. To functionally validate this impaired skin-to-blood migration, future mechanistic studies could employ in vivo models, such as tracking immune cells introduced into the skin of *Rag2-/-* mice during EAE.

We observed an exception to the general finding of failed systemic translation in the cMono, InterMono and CD56-dim NK cells in the skin, where HD showed stronger overrepresentation, consistent with the proportional rise of these populations after UVB in skin (Supplementary Fig. 2).

Interferon-β is an approved disease-modifying therapy against MS and has been proven to be anti-inflammatory [66, 67]. The VDR pathway is enhancing this effect, and interferon-β is enhancing the VDR pathway [68–70]. This positive feedback loop highlights how much HD could benefit more from UVB than RRMS. Therefore, these gene sets were of particular interest and were both found to be strongly represented in HD after UVB irradiation.

Similarly, we observed a stronger response in IL-2/STAT5 signaling, Th1 and Th2, as well as Th17 cell differentiation pathways. IL-2, acting through STAT5 signaling, exerts immunomodulatory effects and plays a key role in regulatory T-cell homeostasis [71–73]. Th2 is considered protective in the context of MS, and shifting the immune response from a Th1 to a Th2 phenotype, as seen with IFN-β treatment, is associated with reduced disease activity [74, 75]. We showed this in the lowered T helper cell differentiation, such as Th2 in blood and Th1 or Th22 in skin (Fig. 1M, N, U). This highlights the far-reaching immunomodulating effects of UVB in the body. With the inhibited response of those genes, regulating inflammation in the blood and potentially the CNS, RRMS patients would need more than UVB or more of it to receive regulatory spillover that could protect against systemic autoimmunity. Nonetheless, both HD and RRMS individuals exhibited a comparable reduction in CSF immune signaling (Fig. 3A–B). Notably, CD8 TRM cells in RRMS showed interferon–β–associated transcriptional changes, adopting a more regulatory and less MS-like profile. The downregulation of *MIF* and *CD99* signaling further supports these as potential therapeutic targets (Figs. 4H and 4M) [44, 76, 77].

We next addressed why MS patients have an impaired reaction to UVB in the blood. It could be, as previously mentioned, a migration-associated problem, wherein UVB-induced cells fail to migrate into the blood, or a problem of reduced VitD. With higher total VitD serum levels MS cell-cell interactions reached an increase after UVB, similar to HD at low VitD serum levels (Fig. 4K). Therefore, higher supplementation with VitD may be key to reach systemic transcriptional changes similar to HD, as it also showed reduced disease activity in a recent study with 100.000 IU/week [25]. VDR is continuously expressed by myeloid cells such as classical monocytes and mDCs and increases upon VitD exposure. However, significant upregulation occurs exclusively in the blood and skin of HD, whereas in MS patients, this significant increase is limited to the skin (Fig. 3D) (Supplementary Fig. 6A). We also observed smaller changes in VitD levels following UVB exposure in MS patients (Supplementary Fig. 8). This, combined with the impaired responsiveness of myeloid cells to VitD stimulation in MS, highlights the diminished beneficial impact of UVB in these patients.

Exposure to sunlight or specifically UVB radiation not only provides immunomodulatory benefits but also carries risks such as skin cancer and accelerated ageing [15]. Nonetheless, analogous to physical exercise, where transient muscle inflammation and injury ultimately support beneficial immune homeostasis, moderate and regular exposure to sunlight has been demonstrated to protect against autoimmune disease activity and severity [8, 11, 78, 79]. Intermittent or prolonged absence of sunlight exposure could disrupt immune homeostasis. Although VitD supplementation may partially compensate for this gap, MS patients, who exhibit a higher prevalence of VitD-related single-nucleotide polymorphisms, might have a diminished capacity to adapt under such circumstances adequately [80].

Complementing our transcriptional analysis, we employed TCR sequencing across the skin, blood, and CSF compartments, offering unique insights into the migratory potential and distribution patterns of T cells in vivo. While single-cell TCR sequencing inherently limits repeated capture of individual clonotypes, each T-cell is sequenced only once, and clonotype detection may be influenced by sampling probabilities—the identification of identical TCR clonotypes across multiple compartments strongly implies directed or systemic migration routes for T-cell populations. Thus, our results provide evidence that at least some T-cell clones initiated in the skin can subsequently be identified within the blood and ultimately the CSF.

Indeed, transcriptomic changes and alterations in cellular communication identified within the CSF (Figs. 3A, B, G and 4M) (Supplementary Fig. 8B-D) further substantiate that UVB-driven modulation initiated in peripheral tissues such as the skin could have direct or indirect influences extending into central compartments critical for MS pathogenesis.

Our findings highlight the potential for UVB and its downstream effects as a non-invasive means of modulating immune function in MS patients and reiterate the concept of the skin-brain axis [81, 82]. This is of clinical significance, given the growing body of evidence linking UVB and VitD levels to MS disease progression and severity. While our study provides valuable insights, several limitations should be acknowledged. Firstly, our study had a relatively small sample size (HD: *n* = 7; RRMS: *n* = 11), which may limit the generalizability and robustness of our findings. Especially in CSF (RRMS: *n* = 3, HD: *n* = 1), results were not compared between disease status but still showed a general response to UVB even in the CNS’s immunity. Larger, well-balanced cohorts will be essential to confirm the reproducibility of observed immune cell alterations. Additionally, the RRMS group included participants receiving various treatments, potentially introducing heterogeneity into our results. Although we addressed these concerns by applying a mixed-model approach in our differential gene expression analyses to account for inter-individual differences, it is noteworthy that despite the uneven group sizes and treatment variability, HD participants consistently exhibited stronger transcriptional responses in blood compared to RRMS, highlighting the robustness of this observed difference. A study like this would not be allowed without additional disease-modifying treatment (DMT) like natalizumab, glatiramer acetate or dimethyl fumarate. Given that they can alter leukocyte trafficking and attenuate skin-to-blood immune surveillance, we performed targeted analyses to address these concerns [83–85]. Treatment-adjusted analysis of overrepresentation overlap showed almost identical effects as without treatment adjustment, and Cellchat score deltas on patient level revealed the treatment naïve participant as the second strongest effect, while effects of dimethyl fumarate-treated participants closer resembled those of HD (Supplementary Fig. 11A&C).

Secondly, the study is conducted during winter to minimize confounding effects of ambient sunlight. However, factors such as individual sun exposure history and genetic differences in Vitamin D metabolism may still contribute to variability in responses. An earlier study also identified that MS patients had a blunted transcriptomic response to VitD supplementation which in addition to the significant difference in VitD serum level increases, could account for some of the impaired effects in this study, especially seen in VitD-related pathways (Fig. 3 & Supplemental Fig. 6) [86].

Additionally, while single-cell RNA sequencing offers a powerful means of identifying immune cell subtypes and functional changes, transcriptomic data alone may not fully capture post-transcriptional modifications and functional protein-level changes. Future studies incorporating proteomic and metabolomic analyses would provide a more comprehensive understanding of immune regulation in response to UVB exposure, as one study highlighted a stabilizing immunity in CIS patients on protein level after UVB irradiation [87].

This study provides novel insights into the immunomodulatory effects of UVB irradiation in RRMS patients and HD. Our findings suggest that UVB exposure not only increases VitD levels but also induces regulatory immune responses that may have implications for MS pathogenesis and therapy beyond VitD. Importantly, we could show that the immune regulation induced by UVB irradiation in the skin translates to systemic transcriptomic changes to a lesser degree in MS and that this reduced translation could be a reason why high levels of VitD might be needed in MS to achieve healthy levels of transcriptomic interaction. The data from this study lay the groundwork for further research needed to validate these observations, to explore potential clinical applications of UVB irradiation therapy, and to better understand VitD supplementation effects in MS.

## Materials and methods

### Patients

Eleven patients diagnosed with RRMS according to the 2017 McDonald criteria were enrolled in the study [88]. Clinical characteristics of the study participants are outlined in the Supplementary Table. Patients included in the study did not exceed an EDSS of 3 or were on interferon beta treatment, as it could mask effects [11]. Additionally, seven healthy participants were recruited. All participants did not receive VitD or any other UV-sensitive dietary supplements 4 weeks before inclusion into the study and did not have a known previous history of UV light sensitivity or any relevant skin diseases or autoimmune comorbidities. Skin types were not classified on an individual level, although the participants did not exceed Fitzpatrick’s Type I – IV. Study participants were recruited between November 2022 and April 2023, months with low environmental levels of UV radiation in Germany (ClinicalTrials.gov ID: NCT05627609).

### Study approval

All experiments and sample collections were carried out according to the Declaration of Helsinki and were approved by the local ethics committee in Muenster, Germany (Ethics Committee of the Board of Physicians of the Region Westfalen-Lippe (2022-439-f-S)). Written informed consent was obtained from all patients prior to inclusion in the study.

### UVB phototherapy

Patients were treated with narrowband UVB phototherapy (311 nm), a proven treatment method for several inflammatory skin diseases e.g. psoriasis [89]. For irradiation, a narrowband-UVB chamber (UVB-311) equipped with Philips TL-01 lamps (PDT 1200; Waldmann, Villingen-Schwenningen, Germany) was used. Before starting the UVB phototherapy, the minimal erythema dose was determined, and 60% of the individual’s minimal erythema dose was chosen as the starting dose [90]. The irradiation dosage was gradually increased by 100 mJ/cm^2^ per session until the development of erythema or until the maximal dose of 900 mJ/cm^2^ was reached. Patients were treated 5× per week for 4 weeks.

### Determining vitamin D levels

Levels of 25(OH)D_3_ were determined in serum samples by luminescence immunoassay with an automated Liaison XL analyzer according to the manufacturer’s instructions (DiaSorin, Dietzenbach, Germany).

Generation of skin biopsies.

Punch biopsies (6 mm) were taken before the start of UVB phototherapy and on the day after the last treatment from the lower back in the morning and stored at 8 °C in MACS Tissue Storage Solution (Milteny) until later procession on the same day. Tissue samples were disassociated and CD45 + cells were isolated following the STAR protocol for cutaneous immune cells [91]. In short, the tissue was minced in a petri dish and processed in a gentleMACS C tube according to the instructions in Miltenyi Whole Skin Dissociation Kit. The suspension was placed on a 70 μm cell strainer (Falcon) and washed with ice-cold wash buffer (PBS + 10% FCS). Suspension was spun down and resuspended in PBS and incubated with Fc-Block and Zombie Aqua (BioLegend) for 10 min. at room temperature (RT). Then anti-CD45 (BioLegend APC) was added and incubated for another 15 min at RT. After FAC-sorting on a FACS Aria III (BD Biosciences), all cells were used for microfluidic partitioning.

### PBMC isolation

Participants’ blood (HD: *n* = 7, RRMS: *n* = 11) was collected before and after UVB intervention. Peripheral blood mononuclear cells (PBMCs) were isolated from leukocyte reduction system chambers by density gradient centrifugation using Lymphopure (BioLegend). Cell suspension was diluted in X-Vivo15 medium (Lonza) to contain 1000 cells/µl and stored on ice until processed later that day.

### CSF cell samples

CSF cell samples were obtained from participants willing to undergo lumbar puncture before and after UVB. Whole CSF was centrifuged, and the cell pellet was resuspended in X-Vivo15 medium (Lonza). CSF cells were stored on ice for immediate processing. Due to the low cell count in the CSF, all isolated cells were used.

### Single-cell RNA sequencing (scRNAseq)

Briefly, single-cell suspensions were loaded on a Chromium Single Cell Controller using the Chromium Next GEM Single Cell 5′ Library & Gel Bead Kit v2 (both from 10X Genomics). Sample processing and library preparation for transcriptome, T-cell receptors and B-cell receptors were performed according to the manufacturer’s instructions using AMPure beads (Beckman Coulter). Samples of individual patients were prepared simultaneously. Sequencing was performed on a local Illumina NextSeq 500/2000 and NovaSeq6000 with a 26-10-10-90 read cycle pattern. Sequencing data was processed using the Cell Ranger pipeline v.7.1.0 (10X Genomics). In brief, raw bcl files were demultiplexed using the Cell Ranger mkfastq pipeline. Subsequent read alignments and transcript counting were done using the Cell Ranger count multi pipeline with standard parameters.

### Flow cytometry

Cryopreserved peripheral blood mononuclear cells (PBMCs) were thawed, washed, and prepared for spectral flow cytometry as previously described [92]. Briefly, following Fc receptor blockade (TrueStain Monocyte Blocker, Biolegend; Cell Blox, Invitrogen) and viability assessment (Live/Dead Blue, Thermo Fisher), cells were surface-stained with a customized panel of fluorochrome-conjugated antibodies targeting CD45, CD3, CD4, CD8, CD19, CD20, CD27, CD31, CD56, CD45RA, CD45RO, CD194 (CCR4), and CD197 (CCR7) in the presence of Brilliant Stain Buffer (BD Biosciences). Complete antibody information, including fluorochrome conjugates, clones, catalog numbers, and Research Resource Identifiers (RRIDs), is provided in Supplementary Table S3. Cells were subsequently fixed and permeabilized (eBioscience Foxp3/Transcription Factor Staining Buffer Set, Thermo Fisher) and stained intracellularly for GATA3. Data acquisition and spectral unmixing were performed on a Cytek Aurora spectral flow cytometer in accordance with previously established protocols.

Following the exclusion of doublets and dead cells, viable leukocytes were identified (Supplementary Figure S5). Total B cells were gated as CD19⁺CD20⁺CD3⁻, from which naive B cells were delineated by the absence of CD27 (CD27⁻). T cells were defined as CD3⁺CD56⁻ and separated into CD4⁺ and CD8⁺ lineages. The CD8⁺ compartment was stratified into central memory (TCM; CD45RA⁻CD197⁺) and effector memory (TEM; CD45RA⁻CD197⁻) subsets. Within the CD4⁺ lineage, the memory fraction (CD45RA⁻CD45RO⁺) was further analyzed to identify Th2 cells, strictly characterized by the co-expression of CD194 and GATA3 (CD194⁺GATA3⁺). A representative gating strategy is shown in Supplementary Figure S5.

### Preprocessing and data integration of scRNAseq

Before integration, all samples were concatenated and assessed for standard quality metrics and the percentage of reads mapped to mitochondrial genes (exclusion at > 6% mitochondrial transcripts) with the help of scanpy(v1.9.4) and AnnData(v0.9.2) in python(v3.10.13). Furthermore, genes mapping to ribosomal proteins (gene symbol starts with “RP”) that are known to confer batch effects were removed, and scrublet (v2.0.3) was used to remove doublets (with a score at > 0.25). Next, standard preprocessing steps including log normalization and identification of variable genes across samples, were performed using scanpy (v1.9.4). Guided by the mean–dispersion plot, we kept the minimal set of highly variable genes that preserved the underlying biological signal, thereby maintaining information content while minimizing noise from non-informative transcripts. For dimensionality reduction and batch effect removal, we used scVI (v1.0.3) to construct a shared space of latent variables using Bayesian variational inference, which was corrected for the nuisance parameters batch (or sample), tissue, UVB exposition, and status. Hyperparameters were set at 256 neurons and 10 latent variables.

### Clustering

Based on the obtained latent space, a neighborhood graph was constructed using scanpy, and clusters were generated by applying the Leiden algorithm. Subsequently, manual annotation with regard to known marker genes was performed to achieve separation between the leukocyte lineages: myeloid cells, CD4 T cells, CD8 T cells, B cells, and NK cells ^52^. As an important part of this study, subsets were generated regarding the lineages, and the basic workflow was repeated. Subsets within lineages were annotated and bundled again to be processed with the semi-supervised model scANVI and represented in a UMAP. For quantification of lineages and lineage subsets, the number of cells within each cluster was calculated as percentage of the total amount of cells within each individual sample. Statistical comparisons of the resulting proportions were performed using two-sided Wilcoxon rank-sum tests.

### Differential gene expression analysis

For differential gene expression analysis, we subsetted the dataset to include specific tissue, lineage, and condition groups. Raw RNA-seq counts were used in EdgeR(v4.2.1) pipeline, where normalization factors were calculated and added to the metadata [93]. A design matrix was constructed to model the effects of UVB exposure, disease status, age, and sex, or additionally VitD serum levels (ng/mL) for VitD-specific analysis. To account for patient-level variability, we structured the data using group_cell() function, grouping cells by patient ID and incorporating normalization offsets. Finally, we applied the nebula(v1.5.3) negative binomial mixed model (NBMM) on raw counts, leveraging hierarchical modeling for single-cell RNA-seq data effects on subject-level random effects to better control false positives while maintaining statistical power [94, 95]. Fold changes were log₂-transformed to improve visual interpretability, and p-values were corrected for multiple comparisons using the Benjamini–Hochberg false-discovery-rate procedure. To summarize UVB-induced transcriptional shifts across donor types and tissues, we computed row-wise averages and binary counts from a gene expression matrix containing significant changes. Genes were scored based on the number of subclusters showing upregulation or downregulation (values > 0 or < 0, respectively). Separate totals were calculated for HD and RRMS subclusters, along with composite delta metrics (delta_RRMS, delta_HD) reflecting opposite-directional regulation (e.g., HD up + RRMS down). Average expression values above and below zero were also calculated per condition. All NA values were set to zero. These metrics were used to rank genes by consistency and magnitude of differential UVB responsiveness between groups.

### Overrepresented gene set analysis

For gene set enrichment analysis (GSEA), differentially expressed genes (DEGs) were extracted from RNA-seq data across multiple conditions. Gene ranking was performed based on log fold change (logFC) and adjusted p-values, with rankings calculated as -log10(padj) × logFC. Enrichment analysis was conducted using GSEApy(v1.1.2) in python, leveraging libraries such as MSigDB Hallmark, GO Biological Process, and KEGG pathways. Both pre-ranked GSEA and overrepresentation analysis (ORA) were applied to identify enriched pathways among upregulated and downregulated genes. Significance was controlled using Benjamini-Hochberg false discovery rate (FDR) correction. ORA was only performed on significantly changed genes (p adjusted < 0.05) by UVB. Differences of overlap in ORA analysis between HD and RRMS were controlled using Fisher test.

### Cell-cell communication analysis

Cell-cell communication between cell type clusters was analyzed using CellChat(v2.1) with default parameters. For comparative analysis, CellChat scores were generated for each patient individually for analysis in correlation with VitD and UVB [96]. Similarly with Cellphonedb(v5.0.1) we calculated the total sum of significant inferred interaction of all lineage subcluster [97].

### T-cell receptor analysis

TCR sequencing data from multiple T-cell samples were preprocessed and integrated using the scRepertoire package (v2.0.0). TCR contig lists were combined using the combineTCR() function, specifying sample identity and retaining cells with multiple clonotypes (removeMulti = TRUE, filterMulti = FALSE). These were subsequently merged with single-cell transcriptomic data using combineExpression() function, enabling clonotype annotation at the single-cell level.

To identify clonotypes shared across specific tissues, we first defined source tissue samples before UVB irradiation and post-irradiation target tissue as *positive groups*, and pre-irradiation target tissue as the *negative group*. Clonotypes shared between both positive groups but absent in the negative group were determined by intersecting unique clonotype nucleotide (CTnt) sequences across tissues, excluding any clonotypes present in negative group. Filtered cells expressing these uniquely shared clonotypes were further analyzed to assess lineage-specific redistribution. Cells with “NA” clonotype annotations were removed, and a source-target label was assigned to all retained cells. The proportion of shared clonotype-bearing cells detected in blood after-UVB relative to their frequency in the skin pre-UVB was calculated on a per-patient basis, stratified by CD4 and CD8 T-cell lineages. These values were normalized by the total number of matching lineage cells in the skin (pre-UVB), excluding entries with “NA” clonotypes. Statistical comparisons of the resulting proportions were performed using two-sided Wilcoxon rank-sum tests.

### B-cell receptor analysis

B-cell receptor analysis was performed using R 4.3.0., the Immcantation framework and its Docker container [98, 99]. In short, productive BCR nucleotide sequences were annotated using IgBLAST, clustered using Scoper, germline-annotated using Dowser, and quantified in terms of somatic hypermutations using SHazaM, all with default parameters.

## Supplementary Information

Supplementary Material 1: Table S1 Clinical data. Table S2 UVB-induced differentially expressed genes summary. Table S3 Antibodies. Figure S1 Lineage proportions across tissues. Figure S2 Lineage subcluster proportions in skin. Figure S3 Lineage subcluster proportions in blood. Figure S4 Lineage subcluster proportions in CSF. Figure S5 Flow cytometric analysis of immune cell populations in PBMCs. Figure S6 Differential Gene expression in HD and RRMS. Figure S7 Vitamin D-dependent overrepresented pathways. Figure S8 Cell-Cell interaction. Figure S9 B-cell receptor analysis. Figure S10 Differentially expressed chemokines. Figure S11 Confounding factors.

Supplementary Material 2.

## Data Availability

To support the results of this study, sequencing data is available on Zenodo (DOI: 10.5281/zenodo.15356526).
