# Supplementary material for: Single-cell analysis of skin and blood reveals systemic immune responses to ultraviolet B and their impairment in MS
Source: J Neuroinflammation. 2026 Jun 3;23:210. doi: 10.1186/s12974-026-03887-z (PMC13288836; doi:10.1186/s12974-026-03887-z)
Supplement: Supplementary file 1 — Supplementary Material 1: Table S1 Clinical data. Table S2 UVB-induced differentially expressed genes summary. Table S3 Antibodies. Figure S1 Lineage proportions across tissues. Figure S2 Lineage subcluster proportions in skin. Figure S3 Lineage subcluster proportions in blood. Figure S4 Lineage subcluster proportions in CSF. Figure S5 Flow cytometric analysis of immune cell populations in PBMCs. Figure S6 Differential Gene expression in HD and RRMS. Figure S7 Vitamin D-dependent overrepresented pathways. Figure S8 Cell-Cell interaction. Figure S9 B-cell receptor analysis. Figure S10 Differentially expressed chemokines. Figure S11 Confounding factors. [file 12974_2026_3887_MOESM1_ESM.docx]

**Supplementary Materials:**

Supplementary Table 1 Clinical data

Clinical data of the study cohort used in analysis

Supplementary Table 2 UVB UVB-induced differentially expressed genes summary

[Supplementary_table_2.csv]

Complete overview of differently expressed genes analysis done by edgeR.

Supplementary Table 3 Antibodies

Antibody staining information for the spectral flow cytometry panel used in this study. All antibodies were validated for use on a Cytek Aurora spectral flow cytometer. RRIDs were obtained from the Antibody Registry (antibodyregistry.org). Intracellular targets are indicated in italic.

| Target | Fluorochrome | Clone | Isotype | Vendor | Cat. No. | Dilution | RRID |
| --- | --- | --- | --- | --- | --- | --- | --- |
| CD45 | APC/Fire 810 | HI30 | Mouse IgG1, κ | Biolegend | 304076 | 01:50 | AB_2860792 |
| CD3 | BV510 | SK7 | Mouse IgG1, κ | Biolegend | 344828 | 02:40 | AB_2563704 |
| CD4 | PE-Vio770 | REA623 | Recombinant human IgG1 | Miltenyi | 130-113-227 | 17:40 | AB_2726038 |
| CD8 | BUV805 | SK1 | Mouse BALB/c IgG1, κ | BD Biosciences | 612889 | 02:40 | AB_2833078 |
| CD19 | SparkNIR685 | HIB19 | Mouse IgG1, κ | Biolegend | 302270 | 02:40 | AB_2832581 |
| CD20 | BV570 | 2H7 | Mouse IgG2b, κ | Sony | 2111660 | 01:50 | [Not yet listed] |
| CD27 | APC-H7 | M-T271 | Mouse BALB/c IgG1, κ | BD Biosciences | 560222 | 04:20 | AB_1645474 |
| CD31 (PECAM-1) | RB780 | WM59 | Mouse IgG1, κ | BD Biosciences | 569368 | 07:40 | [Not yet listed] |
| CD56 (NCAM) | BUV737 | NCAM16.2 | Mouse BALB/c IgG2b, κ | BD Biosciences | 564447 | 04:20 | AB_2744432 |
| CD45RA | BUV395 | 5H9 | Mouse IgG1, κ | BD Biosciences | 740315 | 02:40 | AB_2740052 |
| CD45RO | SuperBright600 | UCHL1 | Mouse IgG2a, κ | Thermo Fisher | 63-0457-42 | 02:40 | AB_2662466 |
| CD194 (CCR4) | PE-Fire810 | L291H4 | Mouse IgG1, κ | Sony | 2397165 | 04:20 | [Not yet listed] |
| CD197 (CCR7) | PE | REA546 | Recombinant human IgG1 | Miltenyi | 130-119-583 | 07:40 | AB_2655953 |
| GATA3 | BB700 | L50-823 | N/A | BD Biosciences | 566642 | 01:50 | AB_2813884 |
| Live/Dead Blue | — | — | — | Thermo Fisher | L34962 | — | [Not yet listed] |

Supplement Figure 1 Lineage proportions across tissues

**
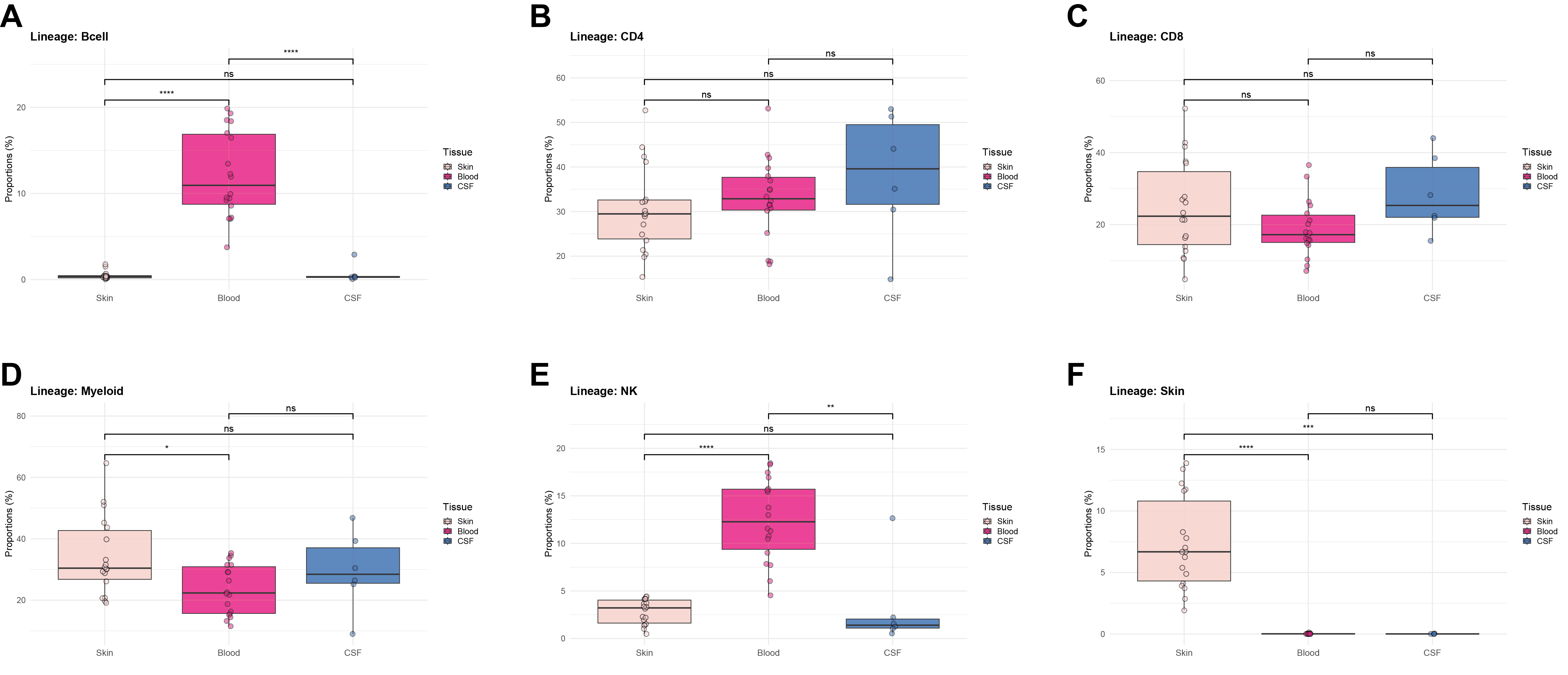
A-F**, Boxplots of mean B-cell (A), CD4 T-cell(B), CD8 T-cell (C), myeloid (D), NK (E), and skin cell (F) proportions in skin (n=36), blood (n=36), and CSF (n=10). Comparisons were done with an unpaired Mann-Whitney U test (* =*p* < 0.05, **=p < 0.01, ***=p < 0.001, ****=p<0.0001). Boxplots show the median (center line), interquartile range (box), and whiskers extending to 1.5× IQR; points beyond are outliers.


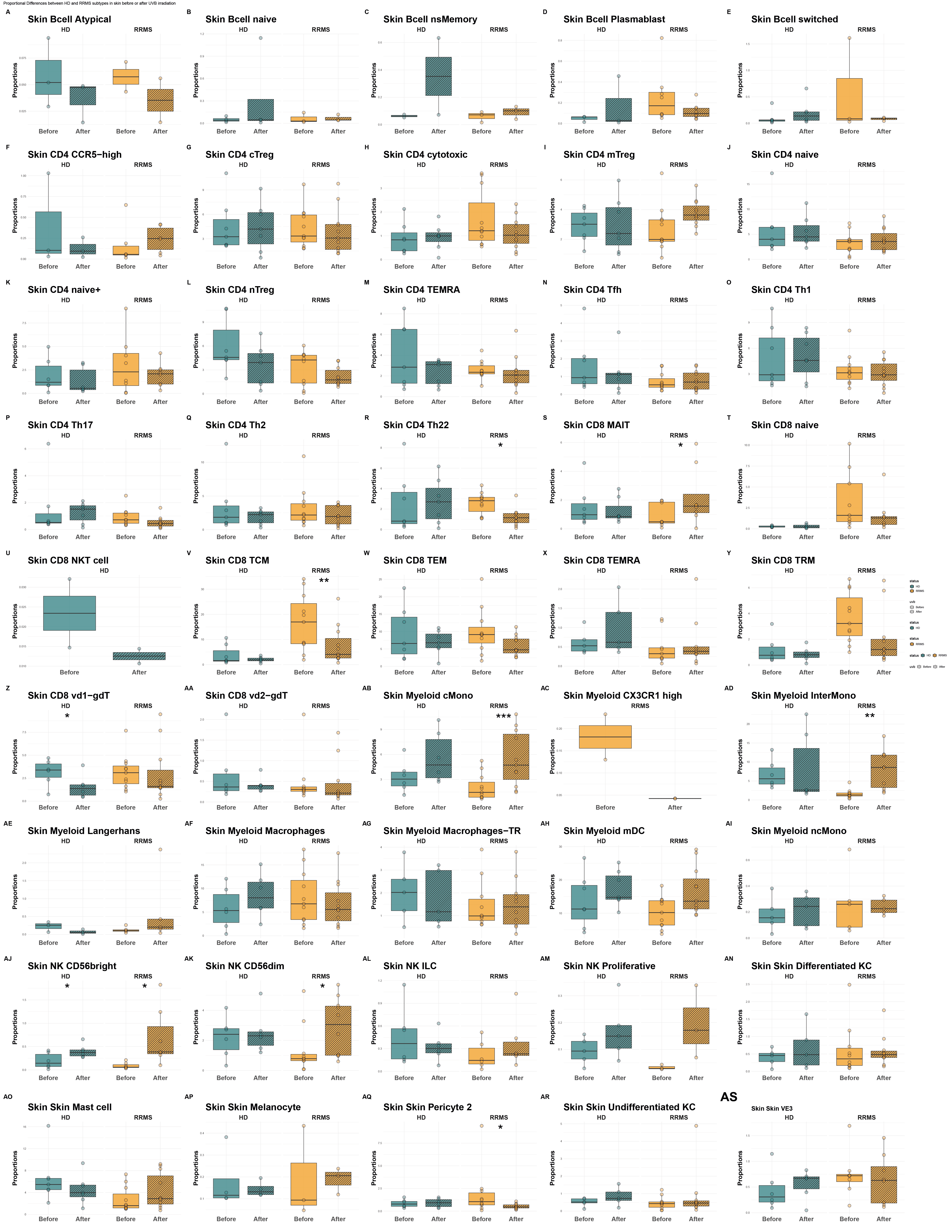


Supplement Figure 2  Lineage subcluster proportions in skin

**A-AS**, Boxplots depicting the proportions of B-cells (A-E), CD4 T-cell (F-R), CD8 T-cell (S-AA), myeloid (AB-AI), NK (AJ-AM), and skin cell (AN-AS) lineage subclusters in HD and RRMS, before and after UVB irradiation, of total annotated skin cells. Comparisons were done with a paired Wilcoxon test (* =*p* < 0.05, **=p < 0.01, ***=p < 0.001, ****=p<0.0001). Boxplots show the median (center line), interquartile range (box), and whiskers extending to 1.5× IQR; points beyond are outliers.


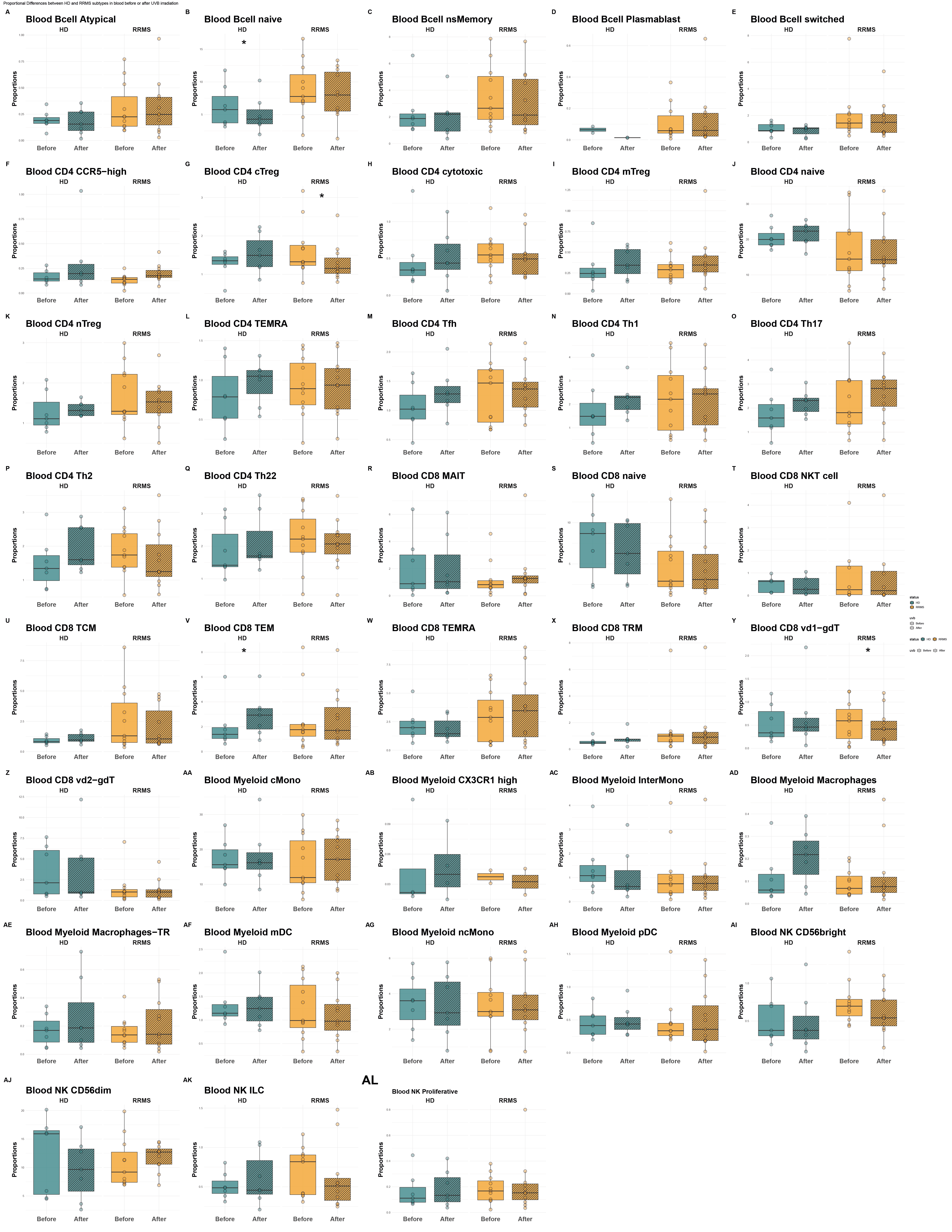


Supplement Figure 3 Lineage subcluster proportions in blood

**A-AS**, Boxplots depicting the proportions of B-cells (A-E), CD4 T-cell (F-Q), CD8 T-cell (R-Z), myeloid (AA-AH), and NK (AI-AL) lineage subclusters in HD and RRMS, before and after UVB irradiation, of total annotated blood cells. Comparisons were done with a paired Wilcoxon test (* =*p* < 0.05, **=p < 0.01, ***=p < 0.001, ****=p<0.0001). Boxplots show the median (center line), interquartile range (box), and whiskers extending to 1.5× IQR; points beyond are outliers.


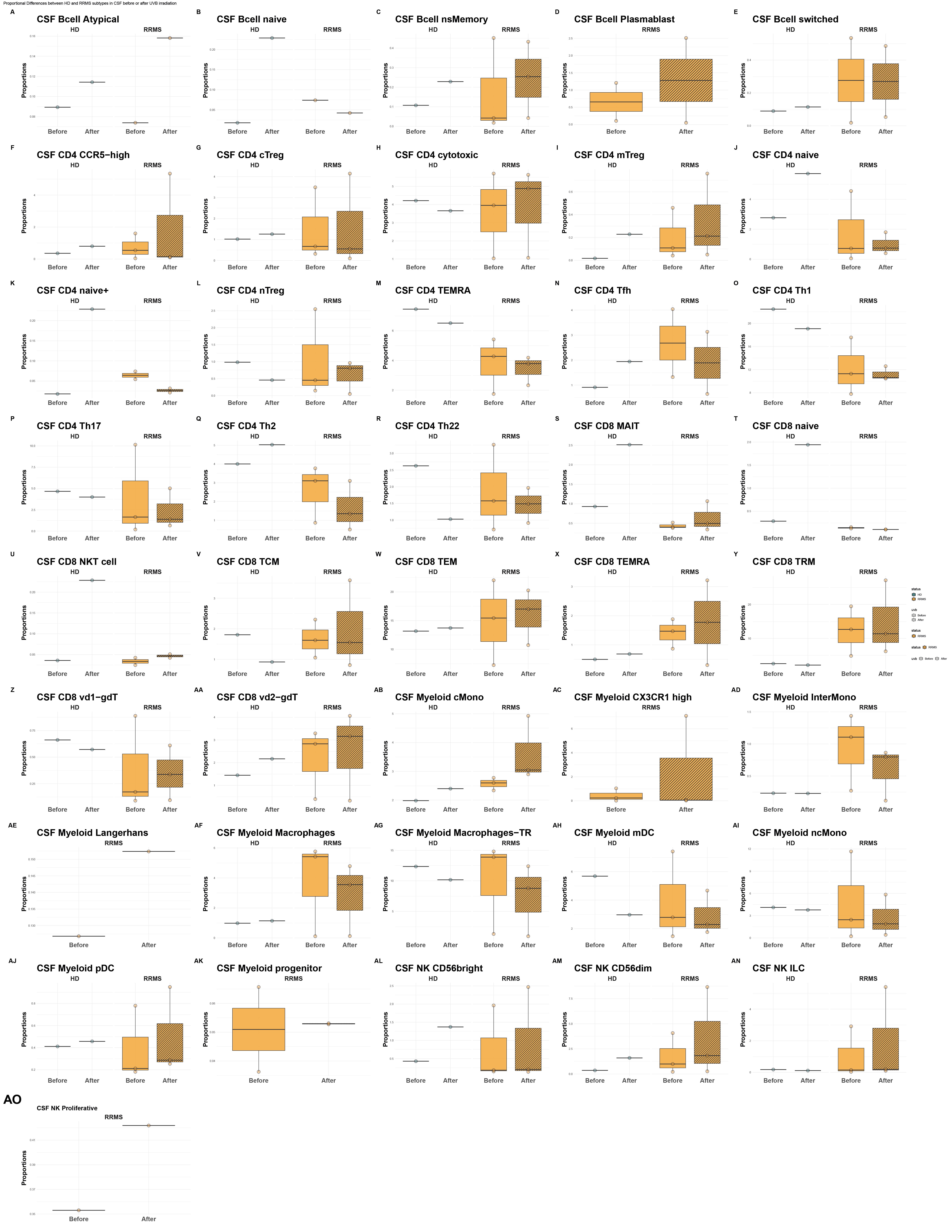
Supplement Figure 4 Lineage subcluster proportions in CSF

**A-AS**, Boxplots depicting the proportions of B-cells (A-E), CD4 T-cell (F-R), CD8 T-cell (S-AA), myeloid (AB-AK), and NK (AL-AO) lineage subclusters in HD and RRMS, before and after UVB irradiation, of total annotated CSF cells. Comparisons were done with a paired Wilcoxon test (* =*p* < 0.05, **=p < 0.01, ***=p < 0.001, ****=p<0.0001). Boxplots show the median (center line), interquartile range (box), and whiskers extending to 1.5× IQR; points beyond are outliers.


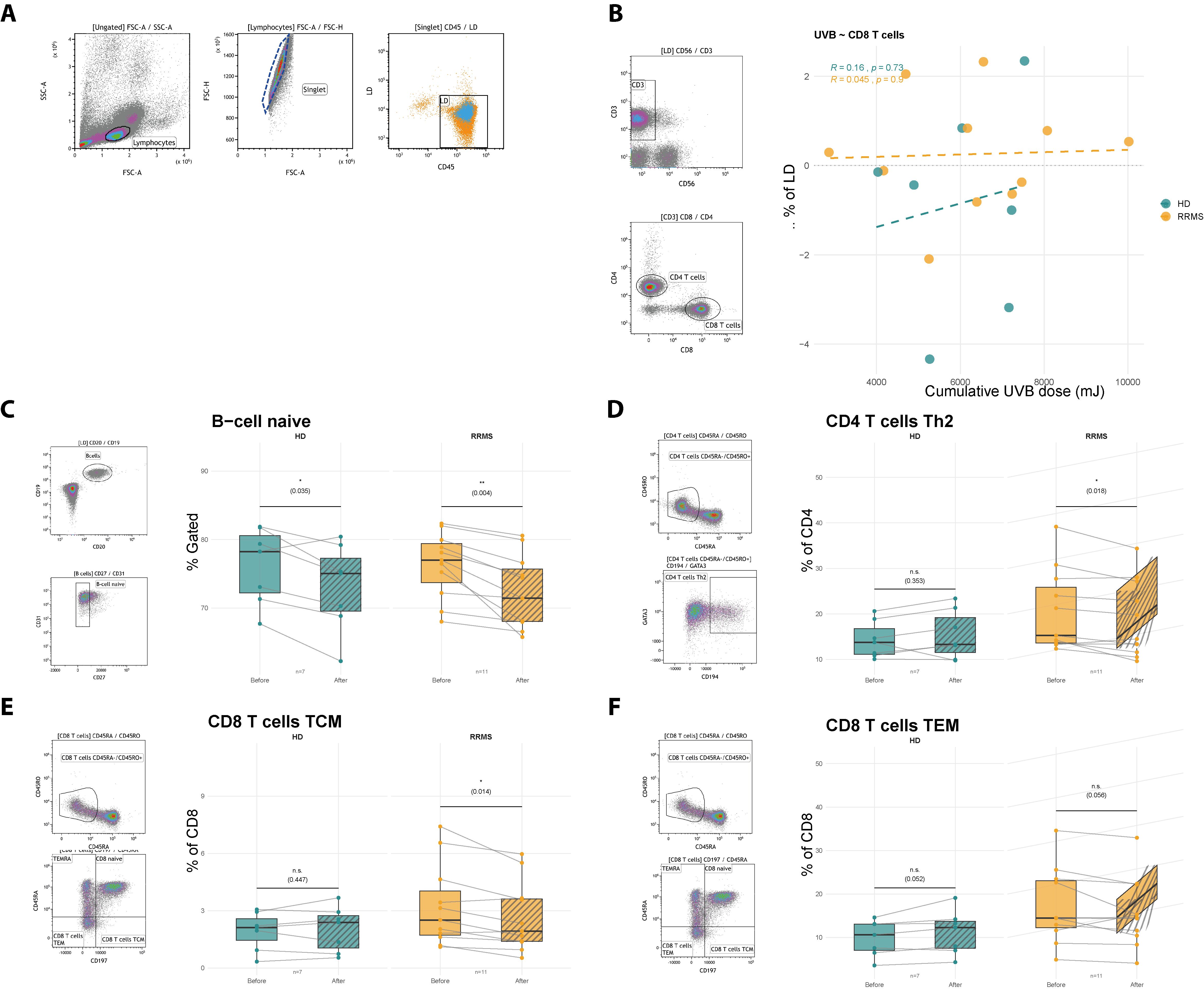


Supplement Figure 5  Flow cytometric analysis of immune cell populations in PBMCs

**A**, Flow cytometry gating strategy isolating viable leukocytes from lymphocytes over singlets to live CD45 positive cells. **B**, Representative gating for total CD8 T cells (CD3⁺CD56⁻CD8⁺) (left) and a scatter plot (right) depicting the differences in CD8 proportions of lymphocytes between before and after UVB irradiation in HD (n=7) and RRMS (n=11) blood against the total UVB irradiation received in mJ. Spearman correlation coefficients were calculated separately for each status group; dashed lines represent linear regressions fitted per group. **C-F**, Representative flow cytometry plots illustrating the gating strategy (left) and boxplots (right) depicting the proportions of **(C)** naive B cells (gated as CD19⁺CD20⁺CD3⁻CD27⁻), **(D)** Th2 CD4 T-cells (gated from the CD4⁺ memory fraction as CD45RA⁻CD45RO⁺CD194⁺GATA3⁺), **(E)** TCM CD8 T-cells (gated as CD8⁺CD45RA⁻CD197⁺), and **(F)** TEM CD8 T-cells (gated as CD8⁺CD45RA⁻CD197⁻) lineage subtypes in HD and RRMS, before and after UVB irradiation. T cells were defined as CD3⁺CD56⁻ prior to lineage separation. Comparisons were made with a paired Wilcoxon test (* = p < 0.05, ** = p < 0.01). Boxplots show the median (center line), interquartile range (box), and whiskers extending to 1.5× IQR; points beyond are outliers.


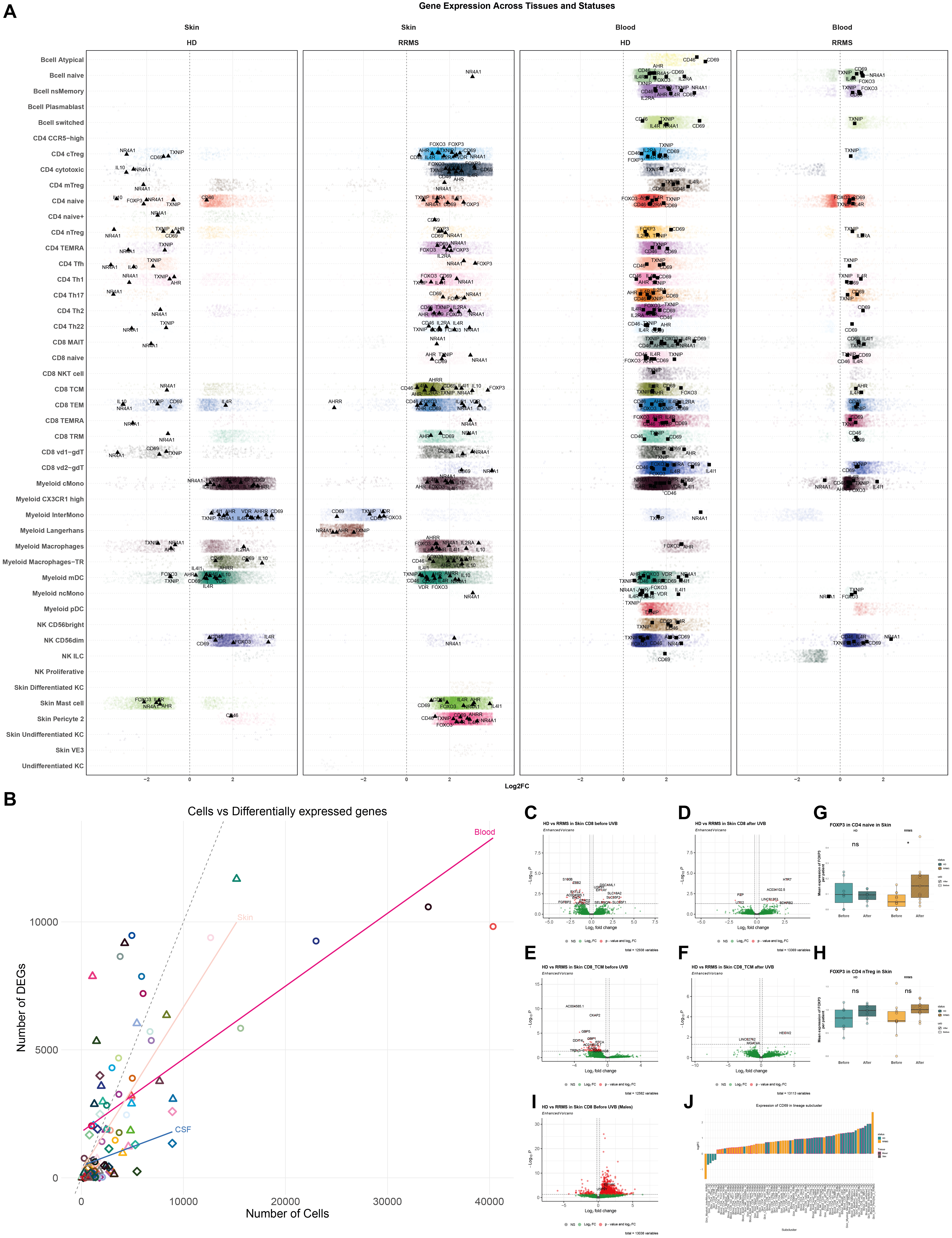


Supplement Figure 6 Differential Gene expression in HD and RRMS

**A,** Log₂FC in gene expression after UVB irradiation for all genes significantly differentially expressed (adjusted p-value < 0.05) as determined by the NBMLM tool *nebula* in HD and RRMS separately. Data are shown for skin (HD: n = 14, RRMS: n=22), blood (HD: n = 14, RRMS: n=22), and cerebrospinal fluid (HD: n = 3, RRMS: n=7). Y-axis was “center-squished”: magnitudes ≤ 2 were linearly stretched two-fold, while larger magnitudes were sign-preserving and logarithmically compressed. **B**, Lineage subcluster plotted at their number of cells against their number of significantly changed genes (p<0.05) as determined by the NBMLM tool *nebula,* lines represent linear regressions fitted per tissue. **C-F**, Volcano plot showing the log2FCs and –log10 of adjusted p-values as determined by the NBMLM tool *nebula* comparing CD8 T cells in the skin before (C) and after (D) between HD and RRMS, and TCM CD8 T cells in the skin before (E) and after (F) between HD and RRMS, dashed line indicates significance threshold (p<0.05). **G&H**, Mean expression of FOXP3 in HD and RRMS patients, before and after UVB irradiation in naive CD4 T cells in skin (G) and naiveTreg CD4 T cells in Blood (H. Comparisons were done with a paired Wilcoxon test (* =*p* < 0.05). Boxplots show the median (center line), interquartile range (box), and whiskers extending to 1.5× IQR; points beyond are outliers. **I**, Volcano plot showing the log2FCs and –log10 of adjusted p-values as determined by the NBMLM tool *nebula* comparing CD8 T cells in the skin before UVB of only male participants. **J**, Gene expression log2 Fold-Changes as determined by nebula analysis for HD and RRMS separately across different lineage subclusters.


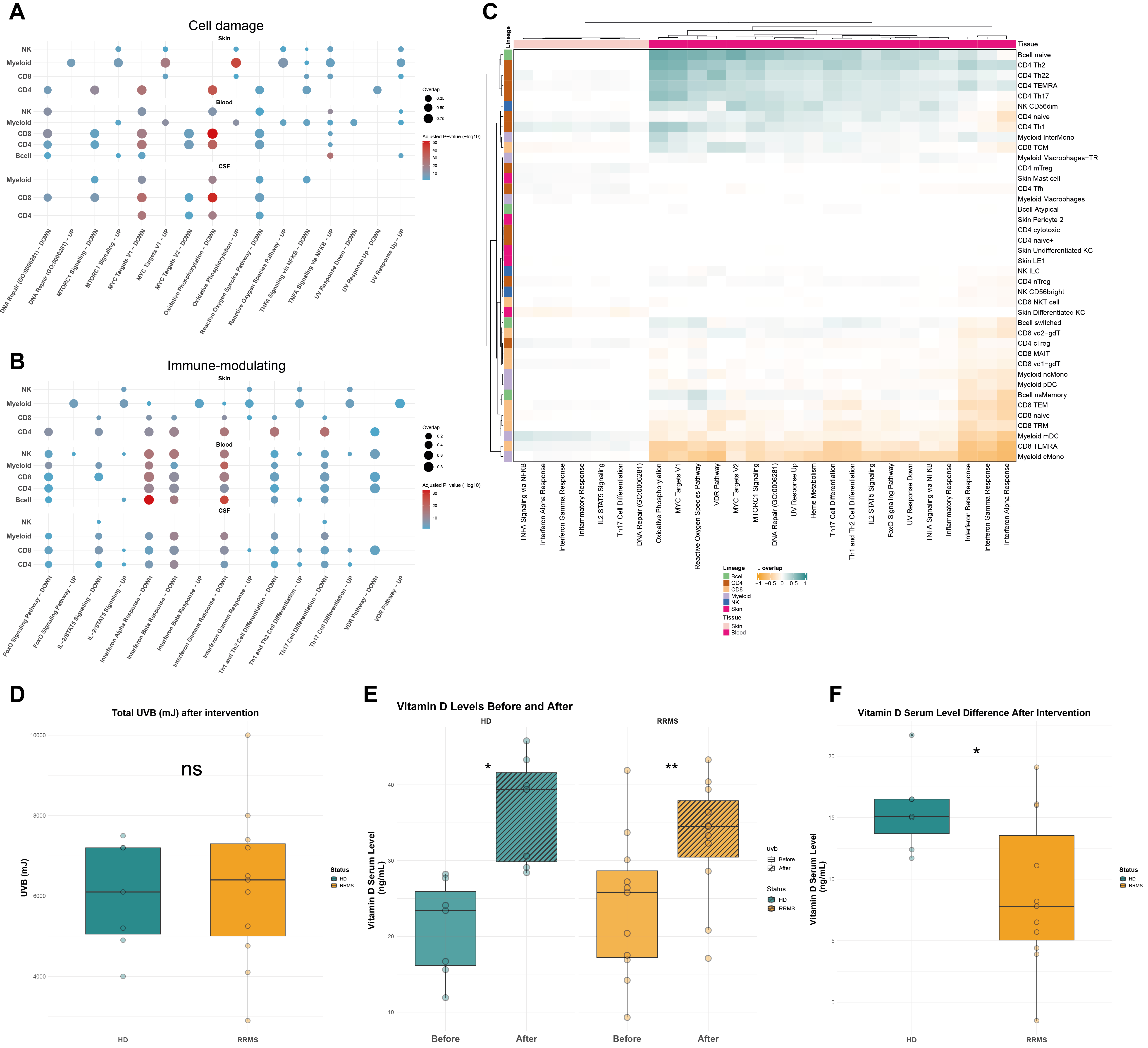


Supplement Figure 7 Vitamin D-dependent overrepresented pathways

**A-B**, Dot plot of overrepresented pathways among significantly changed gene expressions (adjusted p-value < 0.05) after VitD serum level increase in lineages across skin, blood, and CSF. **C**, Heatmap of significant (p <0.05) differences, as determined by Fisher’s exact test, in overlap of only downregulated overrepresented pathways between HD and RRMS (HD-RRMS) in all lineage subclusters from skin and blood after VitD serum level increases, and adjusted by UVB clustered with *ward.D2*. **D**, Boxplots of total UVB irradiation received per patient in mJ in HD (n=7) and RRMS (n=11). Comparisons were done with an unpaired Mann-Whitney U test (* =*p* < 0.05). Boxplots show the median (center line), interquartile range (box), and whiskers extending to 1.5× IQR; points beyond are outliers. **E**, VitD serum levels in ng/mL per patient before and after UVB irradiation in HD (n=7) and RRMS (n=11). Comparisons were done with a paired Wilcoxon test (* =*p* < 0.05, **=p<0.01). Boxplots show the median (center line), interquartile range (box), and whiskers extending to 1.5× IQR; points beyond are outliers. **F**, Differences of VitD serum levels in ng/mL after UVB irradiation between HD (n=7) and RRMS (n=11). Comparisons were done with an unpaired Mann-Whitney U test (* =*p* < 0.05). Boxplots show the median (center line), interquartile range (box), and whiskers extending to 1.5× IQR; points beyond are outliers.


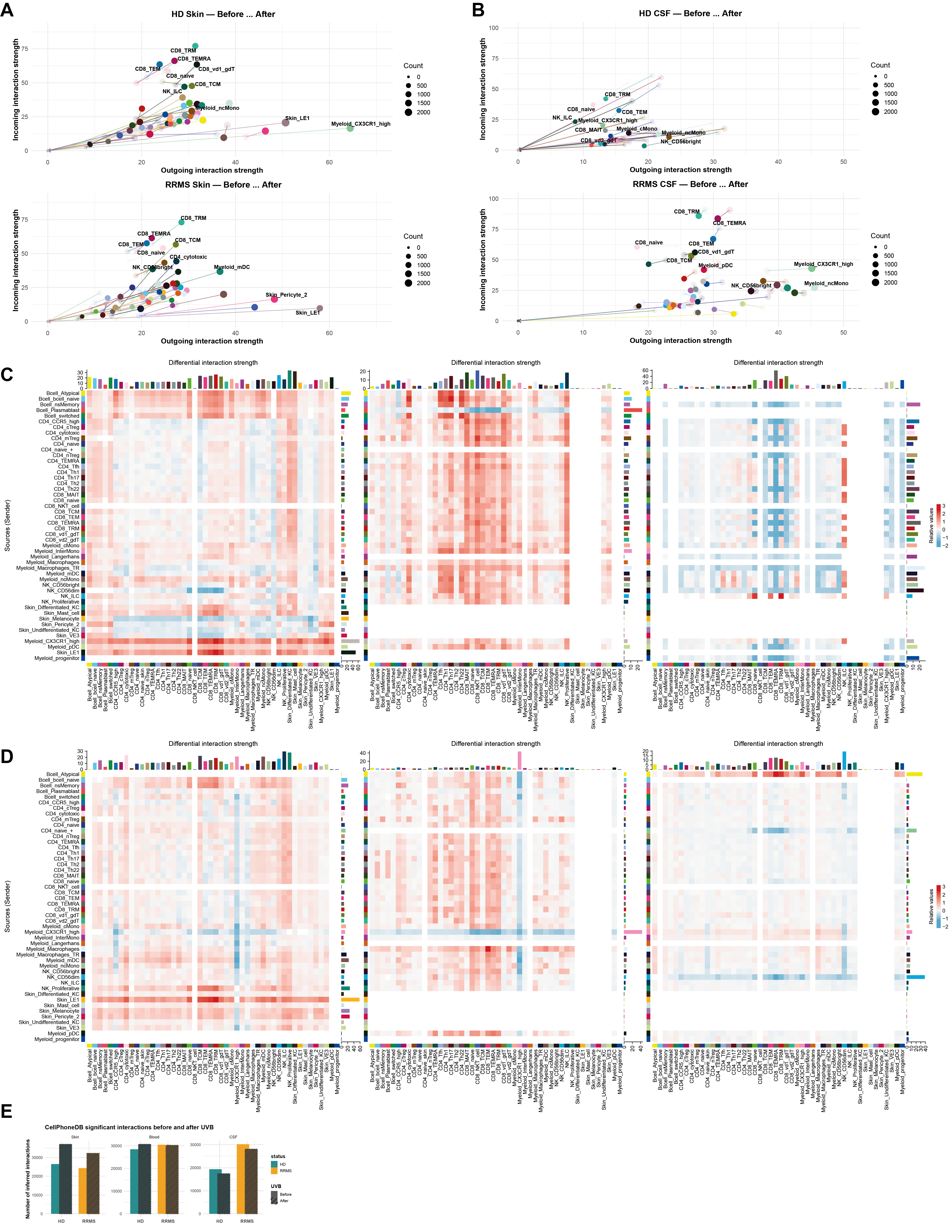
Supplement Figure 8 Cell-Cell interaction

**A**, Scatter plots showing signaling roles (incoming vs. outgoing interactions) of skin-derived cells before and after treatment, comparing healthy donors (HD, top row) to RRMS patients (bottom row),the ten cell types with highest combined signaling strength in the “After” state are labeled. **B**, Scatter plots depicting signaling roles (incoming vs. outgoing interactions) of cerebrospinal fluid (CSF)-derived cells before and after treatment, separately for healthy donors (HD, top row) and RRMS patients (bottom row),the ten cell types with highest combined signaling strength in the “After” state are labeled. **C**, Heatmap showing differences in interaction strength between before and after UVB irradiation of lineage subclusters in skin, blood, and CSF of HD. **D**, Heatmap showing differences in interaction strength between before and after UVB irradiation of lineage subclusters in skin, blood, and CSF of RRMS. **E**, Total number of significant (p<0.05) inferred interactions as calculated by CellPhonedb(v5.0.1) from before and after UVB irradiation in skin, blood, and CSF of HD and RRMS.


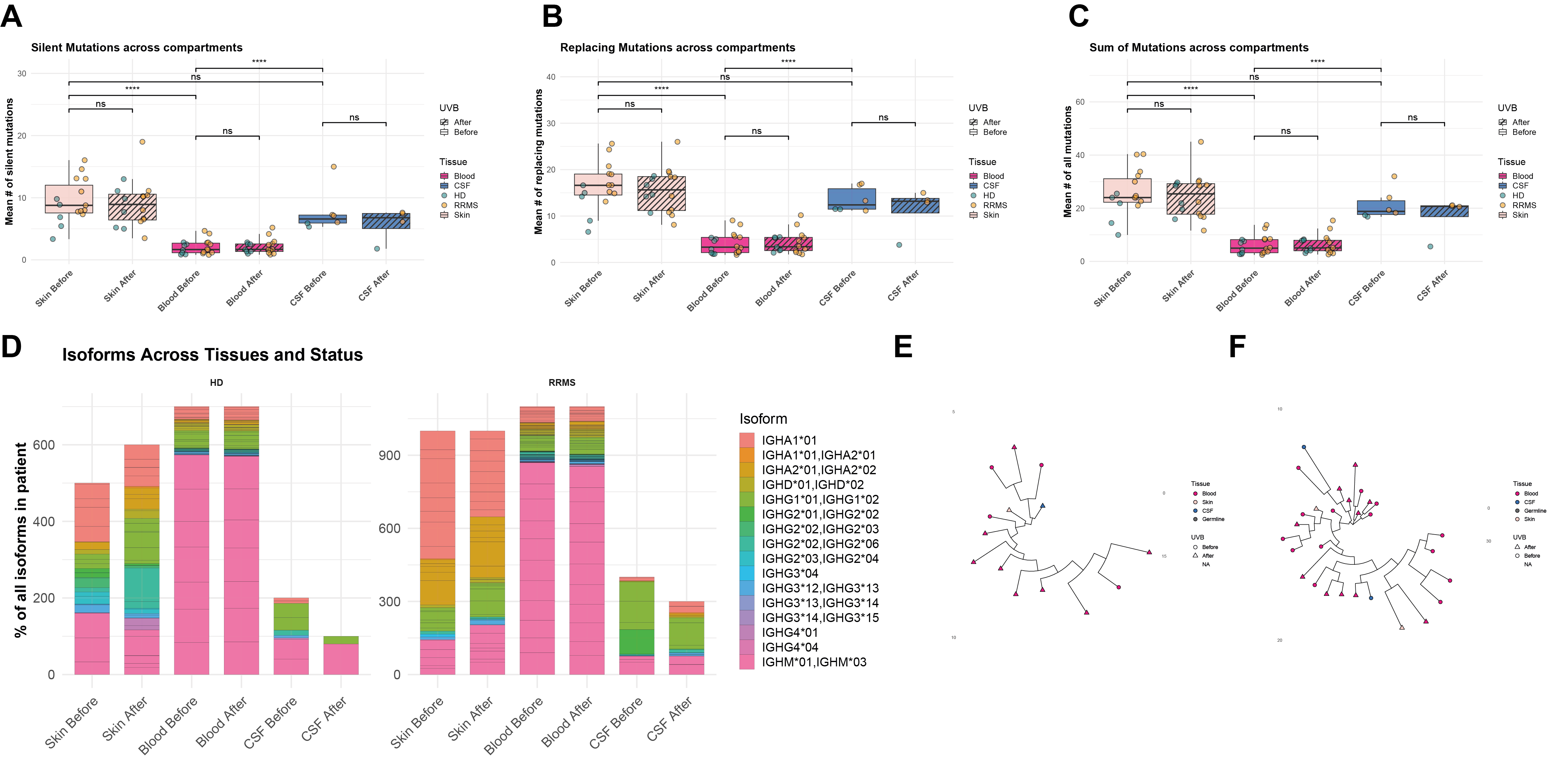


Supplement Figure 9 B-cell receptor analysis

**A-C**, Boxplots depict the mean number of silent (A), replacing (B) and all (C) BCR mutations in per patient across skin, blood, and csf, before and after UVB irradiation. Comparisons were done with an unpaired Mann-Whitney U test (**** =*p* < 0.0001). Boxplots show the median (center line), interquartile range (box), and whiskers extending to 1.5× IQR; points beyond are outliers. **D**, Bar plot of the proportion of each BCR isoform of all isoforms. Each block represents the proportion in one patient, HD or RRMS, in skin, blood, or CSF, before and after UVB irradiation. **E**, Phylogenetic tree of one B-cell germline in HD, depicting BCRs found in skin, blood, and CSF, before and after UVB irradiation. **F**, Phylogenetic tree of one B-cell germline in RRMS, depicting BCRs found in skin, blood, and CSF, before and after UVB irradiation.


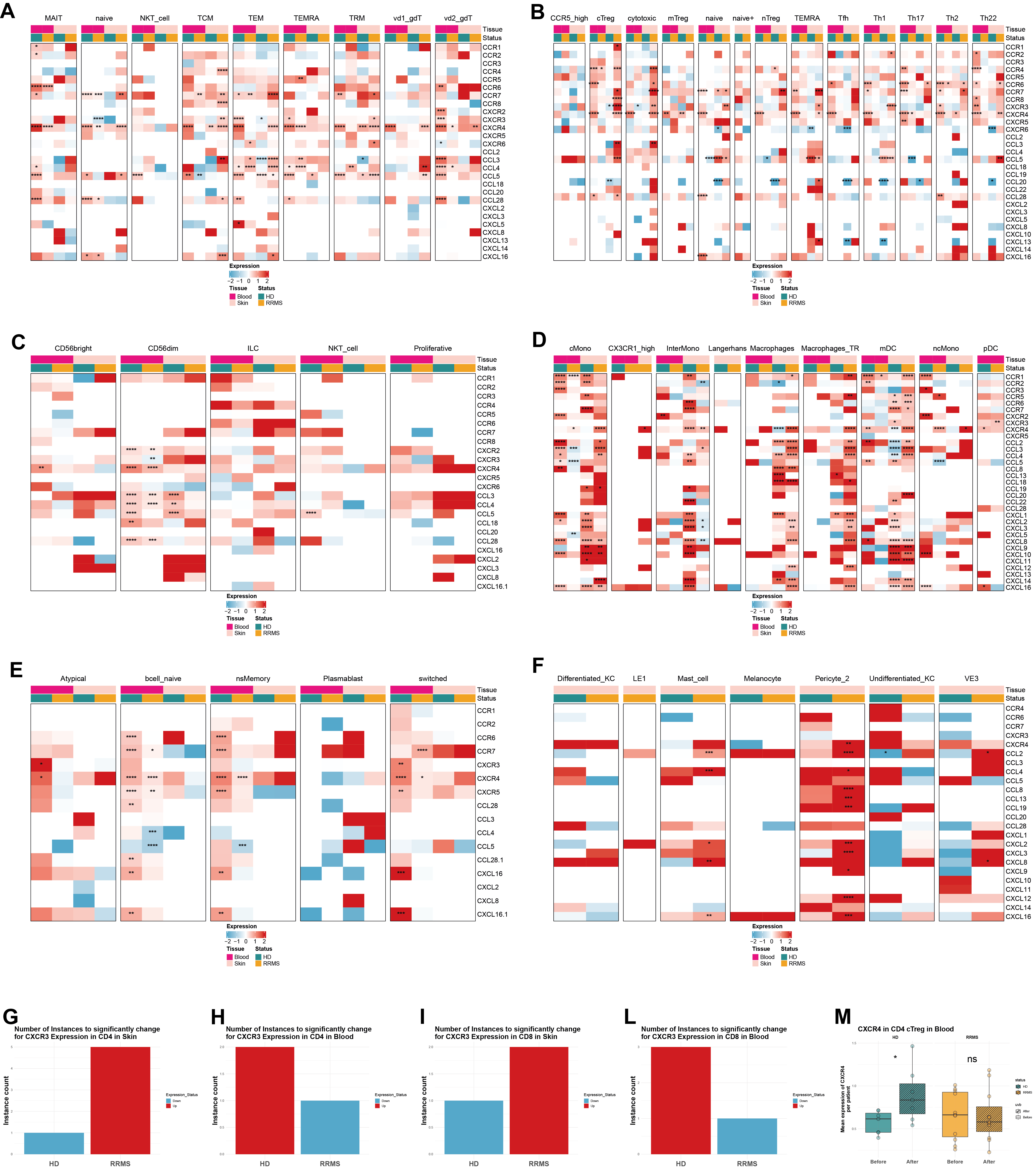


Supplement Figure 10 Differentially expressed chemokines

**A-F**, Heatmap of differentially changed chemokine genes after UVB irradiation in skin and blood from lineage subclusters in HD and RRM of CD8 T cells (A), CD4 T cells (B), NKs (C), myeloid (D), B cells (E), and skin cells (F). The log2FCs are shown as expressions with -2 the lowest and 2 the highest log2FC as was calculated by *nebula*. White colored tiles were an instance of no change in expression. Adjusted p-values are shown as calculated by *nebula* (* =*p* < 0.05, **=p < 0.01, ***=p < 0.001, ***=p<0.0001). **G-L**, Accumulated instances of significant change (p<0.05) in expression of CXCR3 in CD4 T cells in skin (G) and blood (H) and CD8 T cells in skin (I) and blood (L) from HD and RRMS. **M**, Boxplot of mean CXCR4 expression in cytotoxic CD4 Treg cells in blood from HD and RRMS, before and after UVB irradiation, comparison was performed by paired Wilcoxon test. Boxplots show the median (center line), interquartile range (box), and whiskers extending to 1.5× IQR; points beyond are outliers.


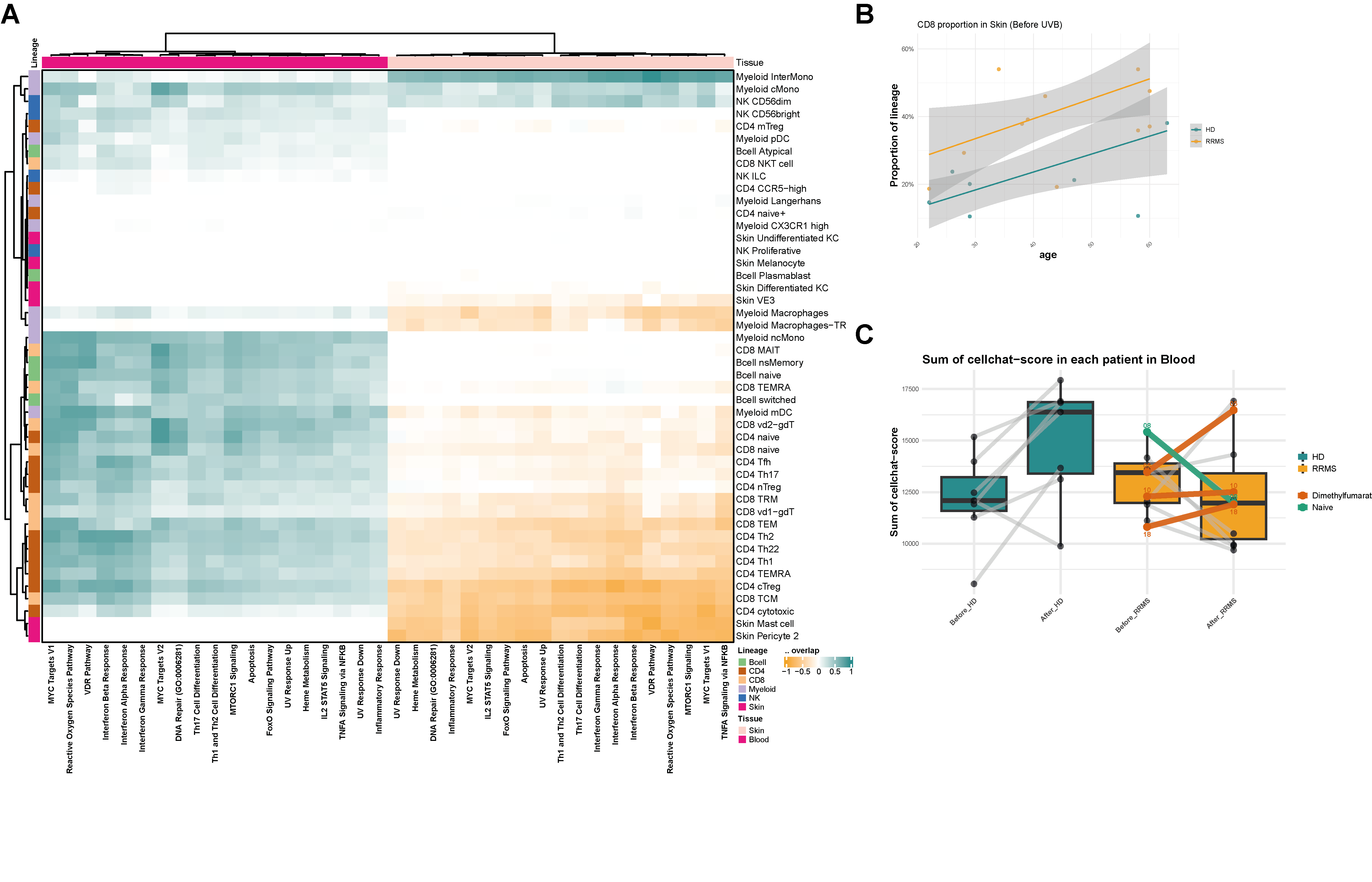


Supplement Figure 11 Confounding factors

**A**, Heatmap of significant (p <0.05) differences after UVB, as determined by Fisher’s exact test, in overlap of only upregulated overrepresented pathways between HD and RRMS (HD-RRMS) in all lineage subclusters from skin and blood adjusted by treatment and clustered with *ward.D2*. **B**, Scatter plot depicting CD8 proportions before UVB irradiation in HD (n=7) and RRMS (n=11) skin against age, dashed lines represent linear regressions fitted per group. **C**, Boxplots depicting the strength of inferred interactions as calculated by CellChat per patient from before and after UVB irradiation blood of HD and RRMS. Highlighted in red are patients on dimethyl fumarate, and blue are treatment naïve.
